# Modelling the quantitative effect of oxygen on the ageing of primed celery seeds

**DOI:** 10.1111/tpj.70066

**Published:** 2025-04-17

**Authors:** Steven P. C. Groot, Paul W. Goedhart, Deborah de Souza Vidigal, Jan Kodde

**Affiliations:** ^1^ Wageningen University & Research Wageningen The Netherlands; ^2^ International Seed Academy Didam The Netherlands; ^3^ Bejo Zaden B.V. Warmenhuizen The Netherlands

**Keywords:** *Apium graveolens*, celery, oxidative damage, oxygen, seed ageing, seed storage

## Abstract

High seed quality is a prerequisite for profitable crop production, but quality declines by ageing during storage. Whereas effects of temperature and humidity are well known, there is limited knowledge on the effect of oxygen. Here, we report on the quantitative effect of oxygen on seed ageing. Primed seeds from celery (*Apium graveolens*) were used as a model, because of their relatively short shelf life. The seeds were stored for up to 7 years at combinations of four relative humidity levels (16, 33, 43 and 60% RH), four temperatures (5, 13, 20 and 30°C) and six oxygen levels (≈1, 5.2, 10, 21, 50 and 99% on volume basis). A strong effect of low oxygen levels was observed at all temperatures and the three lower humidity levels. Modelling the viability data revealed a linear double logarithmic relationship between the oxygen level and the storage time at which the seed lot viability declined to 50% (*p*_50_). The models also showed that each halving of the oxygen level increased seed longevity by around 72%. This implies that reduction of the environmental oxygen level to a level below 1% increased the shelf life of the primed celery seeds by a factor of 11. For seeds pre‐equilibrated at 60% RH, the effect of lowering the oxygen level below 21% was much less pronounced and even absent at 30°C. The large effect of low oxygen level during dry storage of seeds provides opportunities to prolong the shelf life of seeds. Options for practical application are discussed.

## Significance Statement

Effect of different oxygen levels on the shelf life of primed celery seeds at 20°C and 33% relative humidity.

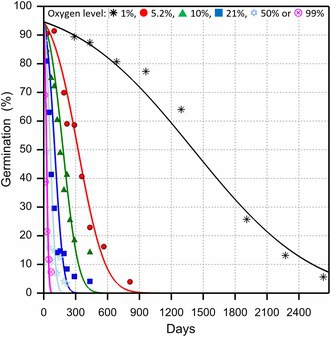

With primed celery seeds, stored between 16 and 45% RH, the oxygen level showed a log‐log linear relation with the rate ageing, with each halving of the oxygen concentration an extension of longevity by around 72%, independent of the storage temperature. Upon storage at around 60% RH these oxygen effects were diminishing.

## INTRODUCTION

Seedling establishment is a critical and sensitive phase in crop production. Vigorous seedlings cope better with biotic and abiotic stresses (Hamman et al., 2002) and seed vigour is a major component in optimising crop yield (Das Gupta & Austenson, 1973; Finch‐Savage & Bassel, 2016; Wimalasekera, 2015). Climate change increases the challenges for seedling establishment, and therefore the need for high‐vigour seeds becomes more urgent. The seed industry puts considerable effort into obtaining high‐quality seeds by optimising seed production, by upgrading treatments such as sorting and priming, as well as storage. The chain from seed production to its use by the farmer can be long with multiple steps of treatments, drying, transport and storage. Unfortunately, seed quality is gradually decreased by ageing during transport and storage, the rate depending on the environmental storage conditions, the species and the physiological status of the seeds. Storage can last from a few months to several years, as with disaster relief programmes, or decades when it concerns *ex situ* conservation in gene banks.

Seed quality declines during dry storage is mainly due to oxidation of lipids, proteins, DNA and RNA (Bailly, 2004; Sano et al., 2015), caused by reactive oxygen species (ROS), and stimulated by elevated humidities and temperatures. Seed ageing can be slowed down by storage under dry, cool and hermetic storage conditions (Ellis & Hong, 2007). Harrington (1972) proposed two rules of thumb: (1) for each 1% increase in seed moisture content the life of seeds is halved and (2) for each 10°F (5.6°C) increase in temperature the life of seeds is halved. Later, the quantitative effects of storage temperature and seed moisture level on seed viability were modelled at a more detailed level (Ellis & Roberts, 1980a; 1980b; Hay et al., 2003), with species‐specific constants that have been estimated for several crops (Society for Ecological Restoration et al., 2023).

In the food industry, it is common to store nuts under controlled anoxic or hypoxic conditions to reduce organoleptic quality loss caused by oxidation, which extends the shelf life of the products (Barden & Decker, 2016; Koelsch et al., 1991). As yet, this is not common practice either in the seed industry or in seedbanks. An occasionally heard misconception is that seeds should need oxygen during storage to stay alive. However, the oxygen requirement for living tissues is related to aerobic respiration, which requires enzyme activity. Respiration enzymes need a minimal amount of water and become only active above around 70% relative humidity (RH) (Groot et al., 2022; Labuza & Dugan, 1971; Walters et al., 2005). At these higher RH levels, the moist seeds will resume anaerobic respiration when brought under hypoxia conditions, resulting in the production of potentially toxic ethanol levels, and eventually, the seeds will die of suffocation (Veselova et al., 2003). Such dying will not happen under dry conditions, as can be deduced from many reported storage experiments where seeds are stored dry in hermetically sealed packages or other types of containers. A considerable number of publications reports that dry seeds can even live longer when stored under reduced oxygen levels, compared with storage in air (Chiu et al., 2003; Gerna et al., 2022; González‐Benito et al., 2011; Groot et al., 2015; Harrison, 1966; Pérez‐García et al., 2007; Roberts, 1961; Roberts & Abdalla, 1968; Schwember & Bradford, 2011; Wang et al., 2018). Low oxygen storage can especially be beneficial for primed seeds, which are generally more susceptible to ageing compared with non‐primed seeds (Fabrissin et al., 2021; Tarquis & Bradford, 1992). The effect of low oxygen on extending seed longevity was found to be reduced at 75% RH compared with 33% RH for primed and non‐primed lettuce and onion seeds (Schwember & Bradford, 2011). More recently, a strong beneficial effect of hypoxia has been shown with *Pinus densiflora* seeds at 11 or 30% RH conditions, where seeds form an intracellular glass, that is, glassy cytoplasm, with high viscosity and restricted molecular mobility, while a beneficial hypoxia effect was lacking with seeds having a fluid cytoplasm at 60 or 80% RH (Gerna et al., 2022).

The likely reason why hypoxia or anoxia can aid in increasing seed longevity is by reducing the production of reactive oxygen species (ROS), generated from the oxygen molecule, for instance by contact with iron or other metal ions in the seed (Apel & Hirt, 2004; Bailly, 2004; Hayyan et al., 2016). With a glassy cytoplasm, enzymatic ROS scavenging is not possible (Gerna et al., 2022), and dry seeds rely on molecular antioxidants, such as tocochromanols and glutathione, that get depleted by oxidation but cannot be regenerated enzymatically under these dry conditions. A negative correlation between seed viability and tocochromanol levels has been shown for seed ageing under dry conditions at 37% equilibrium RH (eRH) for multiple years (Stegner et al., 2022), or after a shorter duration at 35% eRH and an elevated partial pressure of oxygen, while under humid conditions (Controlled Deterioration at 85% eRH and 20°C), the tocochromanol levels did not show a clear decline (Groot et al., 2012; Stegner et al., 2022). Although seed ageing under dry conditions is linked to ROS‐induced damage, the role of oxygen in the seed storage atmosphere seems to be undervalued relative to that of temperature and humidity. As yet, it is even neglected in the most recent standards for gene banks (Food and Agriculture Organization of the United Nations, 2013). This may be due to the observed variation in effectivity, or to a lack of quantitative knowledge on the relative advantage of storage under reduced oxygen levels, compared with the effects of temperature and humidity.

To practically store seeds under hypoxia, it is essential to determine potential advantages over presently used optimal commercial storage conditions (30% RH, 15–20°C with air). For modelling quantitative effects, seed storage should be long enough to also include storage durations significantly reducing seed viability. It was envisaged that such a study with non‐primed seeds would take too long to enable adequate modelling. We therefore used primed celery (*Apium graveolens*) seeds, which may lose commercial quality within a few weeks when stored under ambient conditions (Tanne & Cantliffe, 1989). Earlier experiments showed that anoxia can improve primed celery seed shelf life considerably (Groot et al., 2015). Here, we report on an experiment in which such primed celery seeds were stored at all 96 combinations of four different temperatures (5, 13, 20 and 30°C), four RH levels (15, 33, 43 and 60%) and six oxygen levels (≈1, 5.2, 10, 21, 50 and 99%). Seed viability was recorded at various time points up to 7 years of storage. Observed germination proportions were subsequently modelled as a function of storage time, the three storage conditions (temperature, humidity and oxygen level) and their interactions.

## RESULTS

### Initial analysis of the viability data

**Figure 1:**
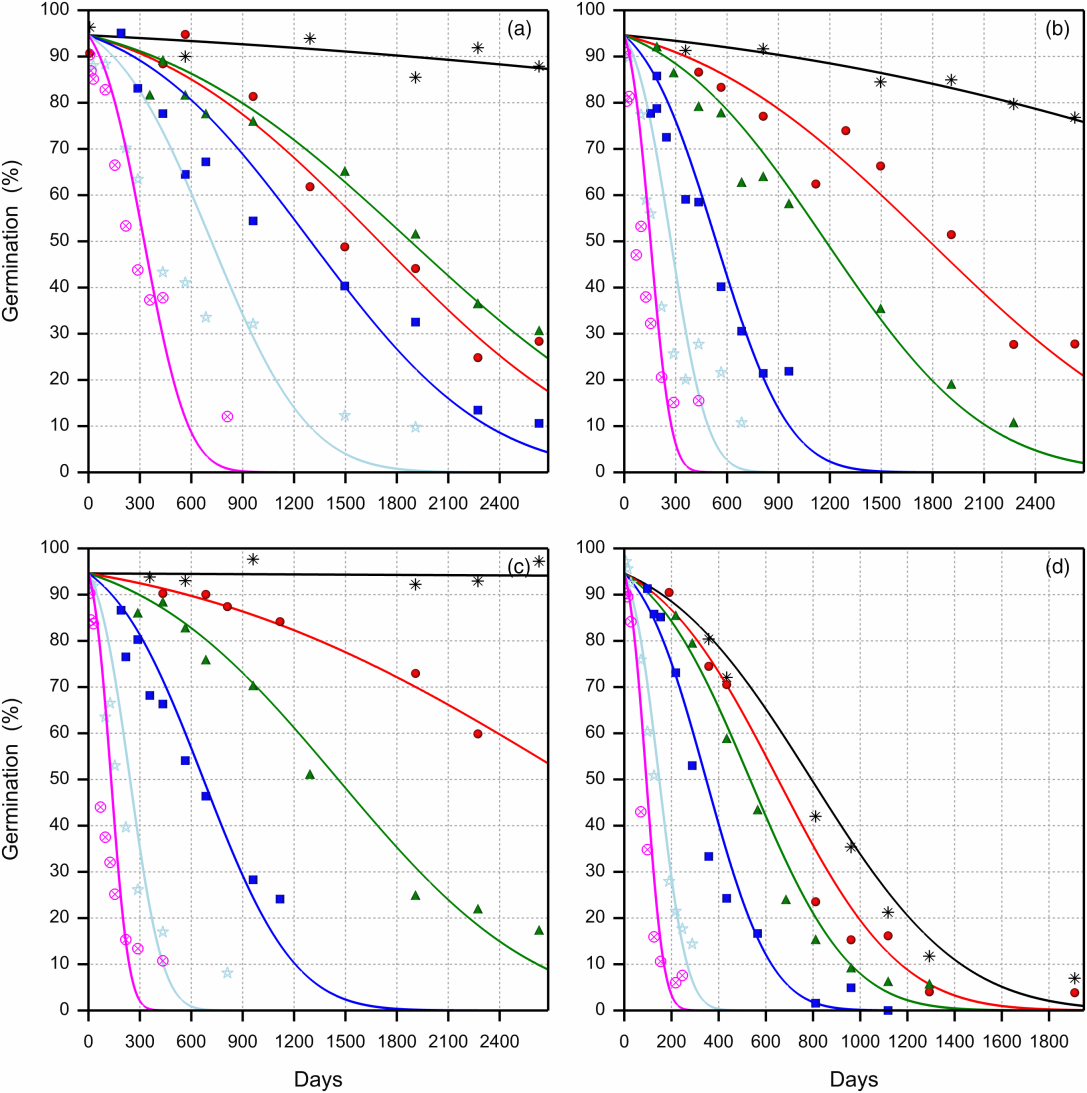
Loss of viability of primed celery seeds during storage at 5°C, four eRH^20^ levels and six oxygen levels. The observed germination data are presented with symbols for the different oxygen levels on a volume basis (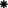 1%, 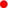 5.2%, 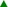 10%, 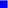 21%, 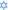 50% and 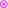 99%). Each symbol represents a single sample. The survival curves were fitted using a probit model according to Roberts and Ellis (1980), with a different slope $\sigma_{S}$ for each storage condition and a single intercept $K_{i}$ representing the initial viability. (a) 16.0% eRH^20^, (b) 32.8% eRH^20^, (c) 43.4% eRH^20^ and (d) 60.3% eRH^20^, where eRH^20^ is the RH in the storage jars after equilibration at 20°C. Note the different X‐axis scales given for the eRH panels.

**Figure 2:**
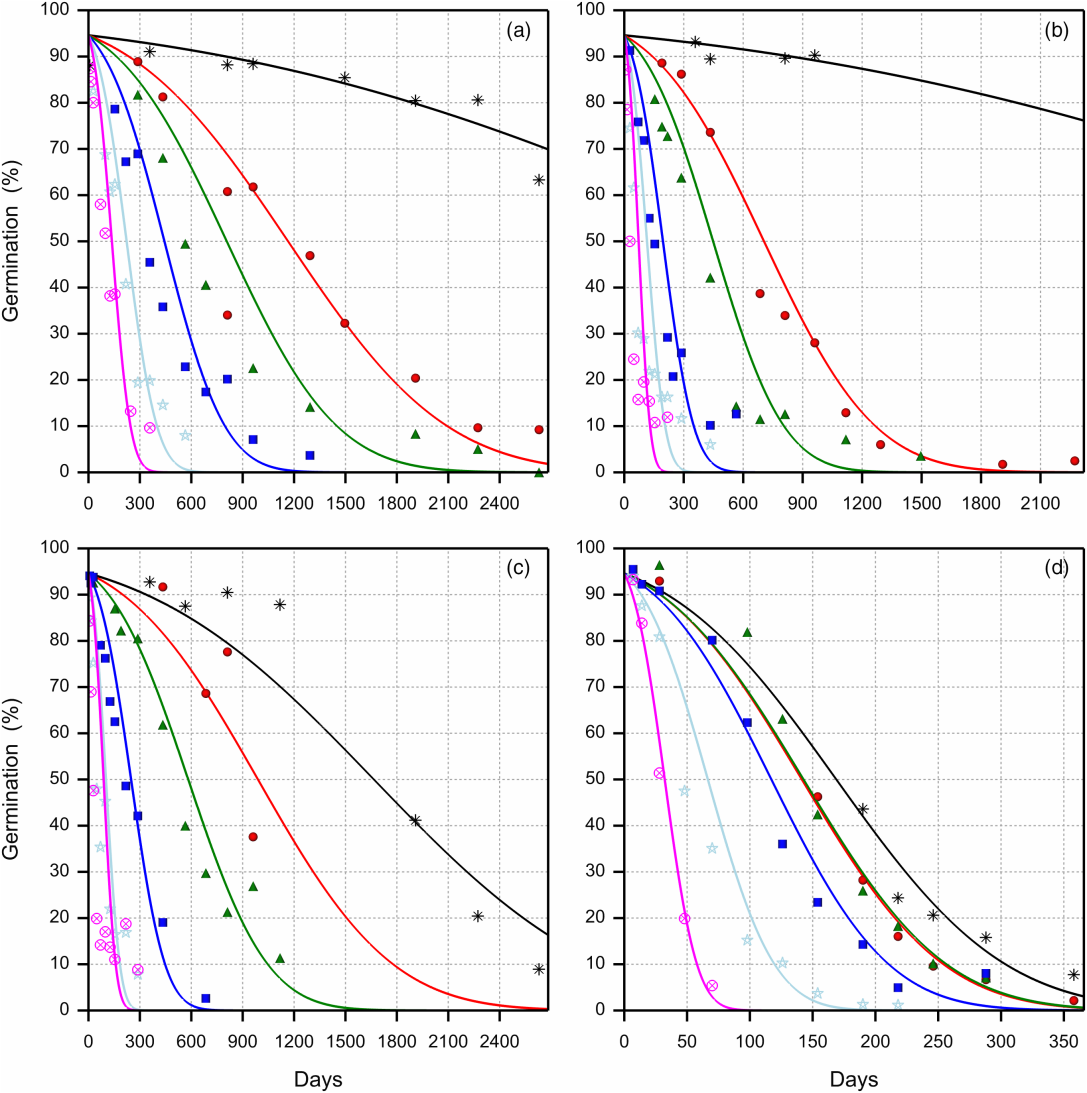
Loss of viability of primed celery seeds during storage at 13°C, four eRH^20^ levels and six oxygen levels. The observed germination data are presented with symbols for the different oxygen levels on a volume basis (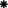 1%, 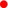 5.2%, 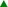 10%, 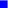 21%, 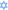 50% and 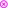 99%). Each symbol represents a single sample. The survival curves were fitted using a probit model according to Roberts and Ellis (1980), with a different slope $\sigma_{S}$ for each storage condition and a single intercept $K_{i}$ representing the initial viability. (a) 16.0% eRH^20^, (b) 32.8% eRH^20^, (c) 43.4% eRH^20^ and (d) 60.3% eRH^20^, where eRH^20^ is the RH in the storage jars after equilibration at 20°C. Note the different X‐axis scales given for the eRH panels.

**Figure 3:**
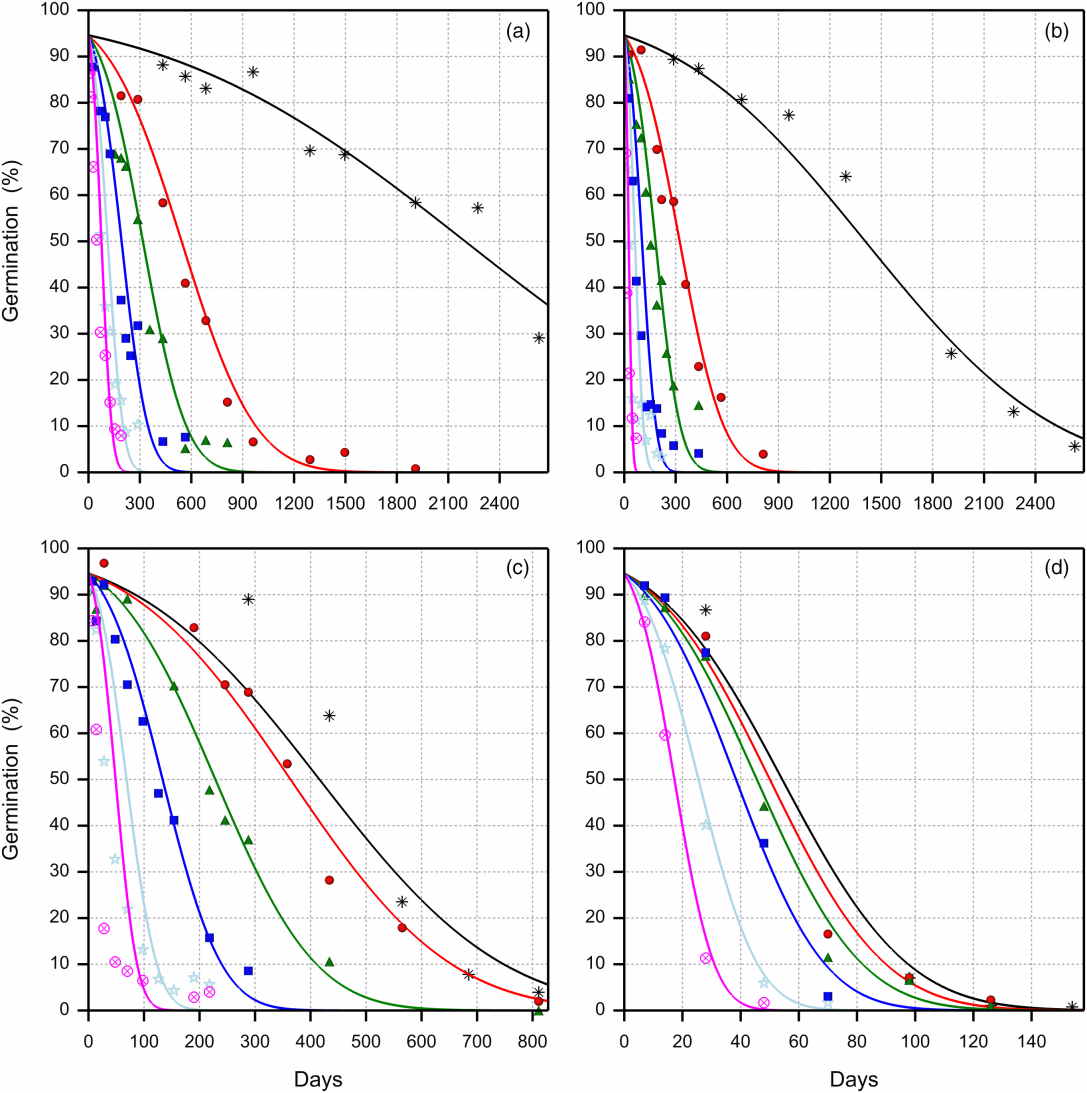
Loss of viability of primed celery seeds during storage at 20°C, four eRH^20^ levels and six oxygen levels. The observed germination data are presented with symbols for the different oxygen levels on a volume basis (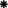 1%, 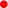 5.2%, 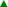 10%, 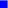 21%, 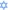 50% and 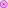 99%). Each symbol represents a single sample. The survival curves were fitted using a probit model according to Roberts and Ellis (1980), with a different slope $\sigma_{S}$ for each storage condition and a single intercept $K_{i}$ representing the initial viability. (a) 16.0% eRH^20^, (b) 32.8% eRH^20^, (c) 43.4% eRH^20^ and (d) 60.3% eRH^20^, where eRH^20^ is the RH in the storage jars after equilibration at 20°C. Note the different X‐axis scales given for the eRH panels.

**Figure 4:**
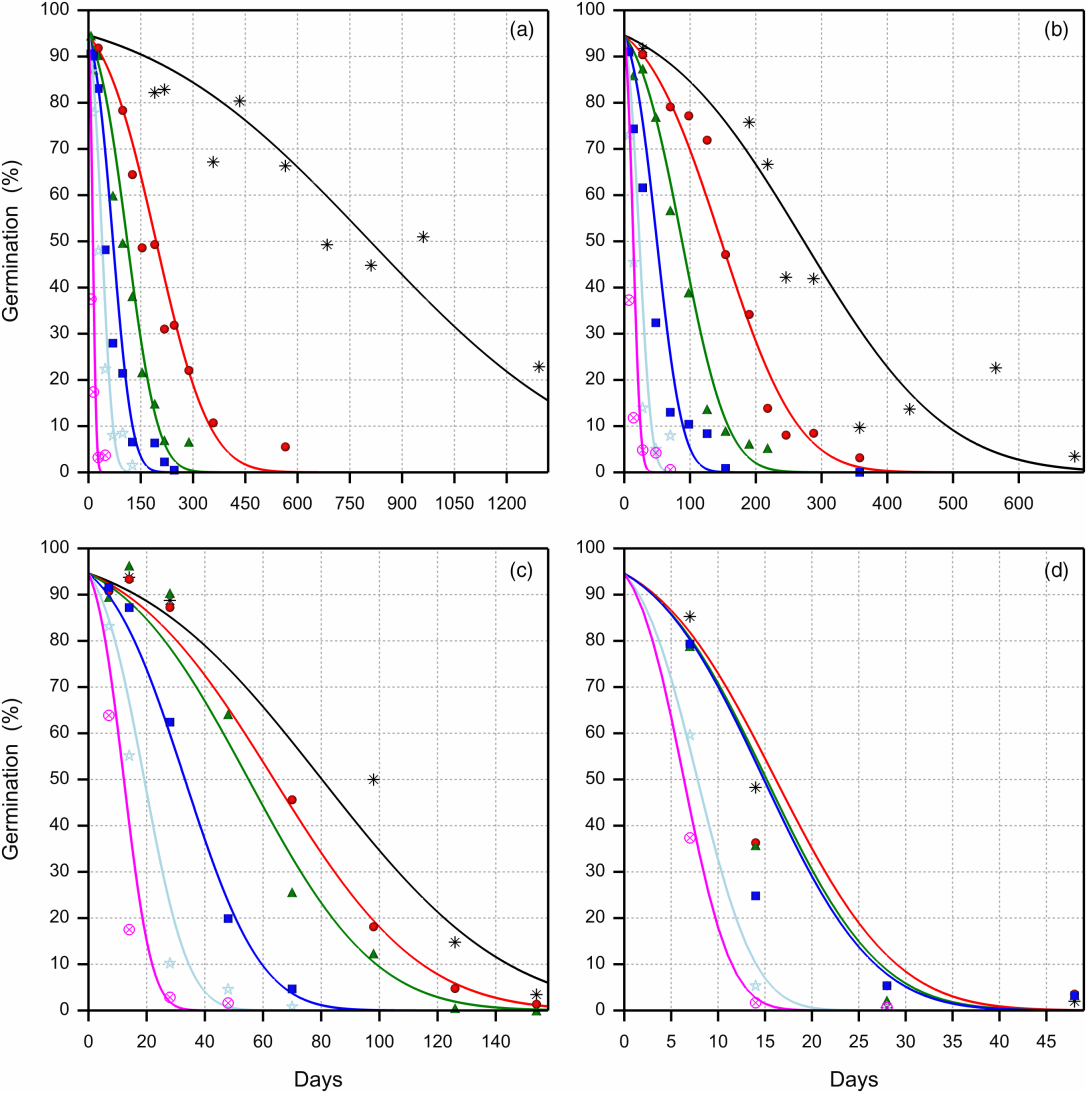
Loss of viability of primed celery seeds during storage at 30°C, four eRH^20^ levels and six oxygen levels. The observed germination data are presented with symbols for the different oxygen levels on a volume basis (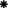 1%, 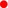 5.2%, 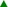 10%, 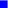 21%, 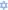 50% and 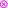 99%). Each symbol represents a single sample. The survival curves were fitted using a probit model according to Roberts and Ellis (1980), with a different slope $\sigma_{S}$ for each storage condition and a single intercept $K_{i}$ representing the initial viability. (a) 16.0% eRH^20^, (b) 32.8% eRH^20^, (c) 43.4% eRH^20^ and (d) 60.3% eRH^20^, where eRH^20^ is the RH in the storage jars after equilibration at 20°C. Note the different X‐axis scales given for the eRH panels.

The raw germination data are provided in supplemental Data S1. As expected, the viability drop followed sigmoidal patterns under the various storage conditions, with faster ageing at warmer temperatures, higher humidity levels and higher oxygen levels (Figures 1, 2, 3 and 4 for storage conditions at 5, 13, 20 and 30°C). In these graphs, the viability curves were fitted using a probit model with a different slope $\sigma_{S}$ for each storage condition.

**Figure 5:**
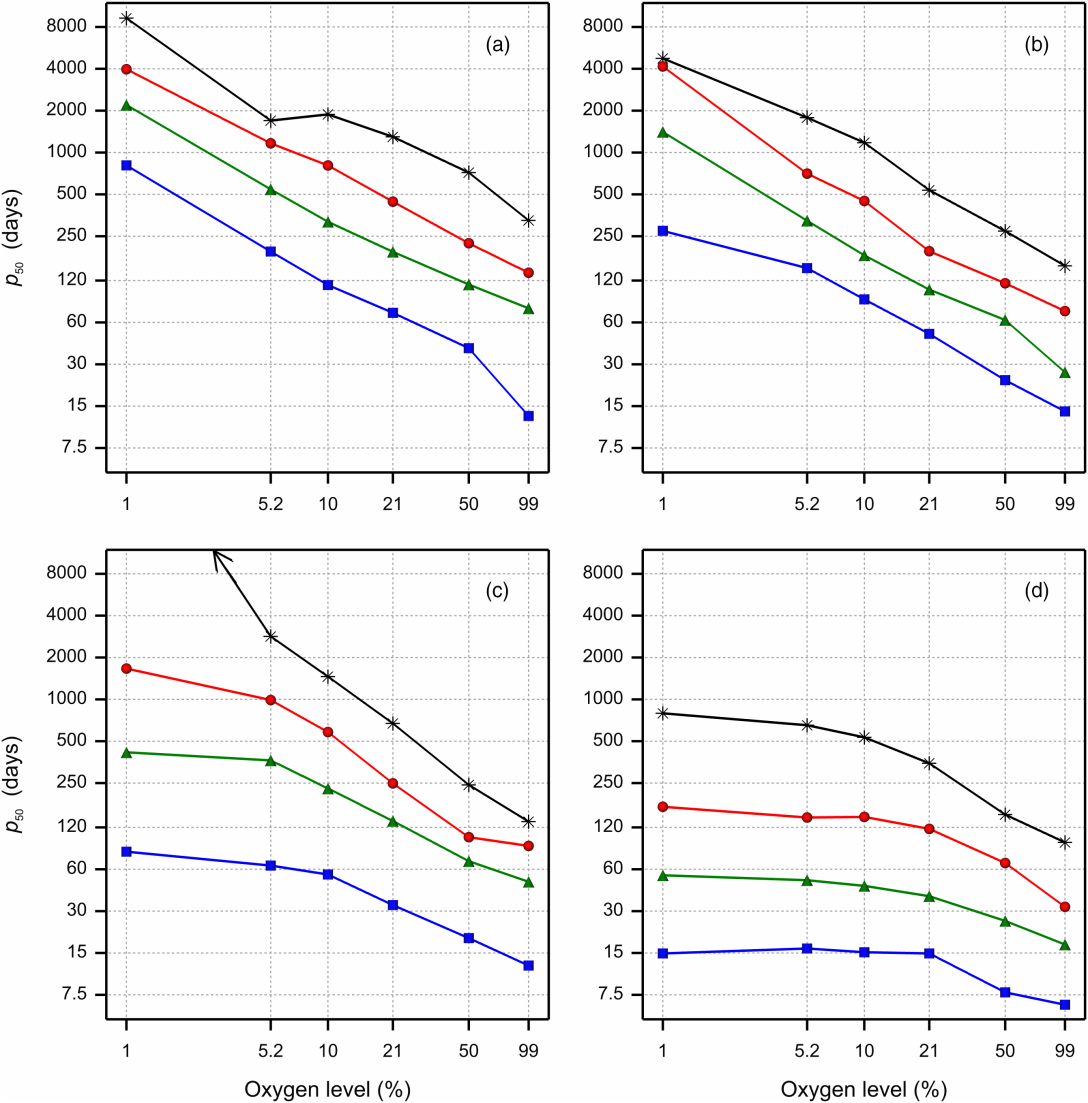
Storage duration when seed lot viability had declined to 50% (*p*_50_) on a log scale as a function of the oxygen level (on a volume basis) on the log scale. The different panels represent the four eRH^20^ storage conditions, each with separate curves for the different storage temperatures (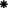 5°C, 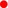 13°C, 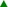 20°C and 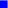 30°C). (a) eRH^20^ is 16%, (b) eRH^20^ is 32%, (c) eRH^20^ is 43%, (d) eRH^20^ is 60%, where eRH^20^ is the RH in the storage jars after equilibration at 20°C. The position of the ‘1%’ oxygen level is less accurate compared with that of the other concentrations, because of the log scale and the related relative higher uncertainty of the actual oxygen level. The arrow in panel c denotes that the estimated *p*_50_ level for the 1% oxygen level and 5°C is beyond 8000 days of storage.

The initial fitted viability curves were summarised by calculating the *p*_50_ value, that is, the estimated storage time when seed lot viability had declined to 50%, for each storage condition. In Figure 5, the *p*_50_ values are plotted against the oxygen level for each eRH^20^ level. This revealed that for three out of the four eRH^20^ levels, log_10_(*p*_50_) is approximately linearly related to log_10_(oxygen %) (logOXY), with more or less parallel lines per eRH^20^ level and temperature. For the eRH^20^ level of 60% (Figure 5d), the curves at all temperatures were flatter, especially at oxygen levels below that of fresh air (21%). Measurements of the oxygen level for the nitrogen‐flushed samples ranged between 0.1 and 1%. The oxygen meter has a ±1% measuring error, according to the manufacturer. Therefore, the actual oxygen levels may have varied between 0 and 2%. Due to the apparent logarithmic oxygen effect a measurement error at the 1% oxygen level has a larger impact than at highest oxygen levels. Therefore, the data for 1% oxygen were not used in the subsequent modelling. Nevertheless, it was observed that the estimated 1% oxygen level clearly extended longevity compared with the 5.2% oxygen level, and the oxygen effect was in the order of magnitude expected with the linear logarithmic trend observed for the higher levels (Figure 5). For the 16, 33 or 43% eRH^20^ treatments and oxygen levels between 5.2 and 99%, the slopes for the *p*_50_ values in relation to the oxygen level are rather similar, both when comparing the different temperatures at each of the individual eRH^20^ levels, as when comparing the eRH^20^ levels. A representation of the data with panels per temperature and a separate line per eRH^20^ level shows again a linear relationship for the 16, 33 and 43% eRH^20^ treatments (Figure S1), with some overlap for 33 and 43% eRH^20^. When the estimated *p*_50_ values were plotted against temperature, with a different panel per eRH^20^ level and a separate line per oxygen level (Supplemental Figure S2), the decline in *p*_50_ with increasing temperatures, could be observed more clearly. These curves are also rather linear, and parallel for the different humidity levels, indicating a linear temperature effect on log_10_ (*p*_50_), at all four humidity levels, independent of the oxygen level.

The slopes of the curves drawn by plotting *p*_50_ against the oxygen level (Figure 5) gave a rough estimate of the effect of oxygen level on the *p*_50_ value per eRH^20^ and temperature condition. The estimated slopes, excluding the *p*_50_ value for the 1% oxygen levels (Table 1), revealed rather similar oxygen effects for the three driest storage conditions, with an overall average regression coefficient of −0.78. This means that for the three driest conditions, halving the oxygen level during storage increased the shelf life of the primed celery seeds by a factor of around 1.72, that is, (1/2)^−0.78^. At 60% eRH^20^, the oxygen effect on seed ageing was less pronounced. Moreover, for 43% and 60% eRH^20^ values the relative oxygen effect was decreasing with increasing temperatures (Figure S4).

**Table 1:** Regression coefficients per eRH^20^ and temperature for the model log(*p*_50_) ~ log(oxygen) excluding the *p*_50_ values for oxygen = 1%, where eRH^20^ is the RH in the storage jars after equilibration at 20°C

Temperature\eRH^20^	16%	33%	43%	60%
5°C	−0.57	−0.85	−1.06	−0.69
13°C	−0.74	−0.79	−0.88	−0.50
20°C	−0.66	−0.81	−0.70	−0.36
30°C	−0.86	−0.81	−0.58	−0.34
Average	−0.71	−0.81	−0.80	−0.47

### Modelling viability loss per eRH^20^ level

In the previous analyses, the effects of oxygen on seed ageing were modelled separately for each storage condition. This revealed that per RH^20^ value, the log_10_(*p*_50_) could be well described by a regression type model which is linear in temperature (*TEMP*) and linear in log*OXY* (Figure 5; Figure S1). This results in the E&R model given in Equation (1). This E&R model was fitted to the data per eRH^20^ level and subsequently, forward selection was used to see whether the addition of a quadratic effect in *TEMP*, a quadratic effect in log*OXY* and/or an interaction between *TEMP* and log*OXY* should be added to the model (Table 2). Again, the 1% oxygen level was omitted from this analysis. For all four eRH^20^ levels, the model with a separate curve for each of the 20 combinations of oxygen and temperature, that is, the initial model, was significantly better (*P* < 0.001) than the E&R type model with *TEMP* and log*OXY* only.

**Table 2:** Analysis of deviance for forward selection starting with a model with temperature (*TEMP*) and log_10_ (oxygen %) (log*OXY*) only for the various target eRH^20^ conditions, where eRH^20^ is the RH in the storage jars after equilibration at 20°C. A *P*‐value for the Start model is not given since this would require some null model, for example, a constant viability across storage conditions, to compare the Start model with. Such a *P*‐value would be extremely small as can be seen in Figures 1, 2, 3, 4.

eRH^20^ level	Terms	Model	Deviance	Mean deviance	Deviance difference	*F*‐value	*P*‐value
16%	Start	0	2785.96	15.31			
	+ *TEMP* × log*OXY*	1	2718.35	15.02	67.61	4.50	0.035
	+ log*OXY* ^2^	2	2659.11	14.77	59.24	4.01	0.047
	+ *TEMP* ^2^	3	2658.64	14.85	0.47	0.03	0.859
33%	Start	0	4570.86	25.54			
	+ *TEMP* ^2^	1	4371.27	24.56	199.60	8.13	0.005
	+ *TEMP* × log*OXY*	2	4369.41	24.69	1.85	0.08	0.784
	+ log*OXY* ^2^	3	4365.29	24.80	4.12	0.17	0.684
43%	Start	0	4361.28	26.92			
	+ *TEMP* × log*OXY*	1	3703.51	23.00	657.76	28.59	<0.001
	+ *TEMP* ^2^	2	3514.37	21.96	189.14	8.61	0.004
	+ log*OXY* ^2^	3	3506.34	22.05	8.03	0.36	0.547
60%	Start	0	2217.69	17.33			
	+ log*OXY* ^2^	1	1686.42	13.28	531.26	40.01	<0.001
	+ *TEMP* × log*OXY*	2	1229.04	9.75	457.38	46.89	<0.001
	+ *TEMP* ^2^	3	1034.52	8.28	194.52	23.50	<0.001

For storage at 16% eRH^20^ the forward selection with the E&R type model, starting with *TEMP* and log*OXY* only, showed a significant interaction (*P* = 0.035), and on top of that a quadratic log*OXY* effect (*P* = 0.047) (Table 2). The estimated *p*_50_ values for the different models, including the *p*_50_ separately estimated for each storage condition in the initial analysis (Table S1), revealed that the *p*_50_ values are more or less equivalent for the different models, except for one ‘outlying’ combination (*TEMP* = 5°C, *OXY* = 5.2%, also see Figure 1a). Even though the separate model for each combination (temperature and oxygen) fits better, the difference in *p*_50_ values with Model 0 is rather small. This implies that for practical purposes, the simplest E&R type Model 0 would suffice for eRH^20^ 16%. According to this model, at 16% eRH^20^, halving the oxygen level increases the shelf life for each viability level, such as *p*_50_ and *p*_90_, by a factor of 0.5^−0.708^ = 1.63 with standard error 0.025 (Table 3). In this model, the ‘shelf life extension factor’ is independent of the temperature. Dropping the temperature by 5°C increases shelf life by a factor of 10^(−0.0488×−5)^ = 1.75 (±0.022 standard error) while increasing the temperature by 5°C reduces shelf life by a factor 10^(−0.0488×5)^ = 0.57 (±0.007), which is almost halving shelf life. This temperature effect is independent of the oxygen level (Table 2). Shelf life extension factors for other oxygen and temperature effects are given in Data S3.

**Table 3:** Estimates and standard errors (SE) for the ‘best’ E&R type model, indicating the effect of oxygen and temperature at different eRH^20^ levels, where eRH^20^ is the RH in the storage jars after equilibration at 20°C

Parameter	eRH^20^ 16%		eRH^20^ 33%		eRH^20^ 43%		eRH^20^ 60%	
	Estimate	SE	Estimate	SE	Estimate	SE	Estimate	SE
Constant	4.02	0.0398	3.90	0.0558	4.26	0.106	2.98	0.0913
*TEMP*	0.0488	0.00109	0.059	0.00573	0.0577	0.00836	0.0956	0.00478
log*OXY*	0.708	0.0217	0.806	0.0256	1.12	0.0618	−0.274	0.130
*TEMP* ^2^			−0.000450	0.000158	0.000534	0.000176	−0.000534	0.000111
log*OXY* ^2^							0.371	0.0458
*TEMP* × log*OXY*					−0.0184	0.00335	−0.0157	0.00203

For storage at 33% eRH^20^, the analysis of deviance (Table 2) shows that the quadratic term in TEMP (Model 1) is very significant (*P* = 0.005) while the interaction and the quadratic log*OXY* effect are not. Even though the separate model fits better (Table S2), the *p*_50_ differences for Models 1, 2 and 3 with Model 0 are rather small. This seems to imply that for practical purposes the E&R type model 1 would suffice. For Model 1, the estimated shelf life extension factor for halving the oxygen level equals 0.5^−0.806^ = 1.75 (±0.031) (Table 3), again independent of the temperature. Because of the quadratic temperature effect in Model 1, the extension factor for dropping the temperature by 5°C depends on the temperature itself. For starting temperatures of 5°C, 13°C, 20°C and 30°C the shelf life extension factor for reducing the temperature by 5°C is respectively 1.92 (±0.110), 1.77 (±0.053), 1.65 (±0.024) and 1.48 (±0.057) (Data S3). This is independent of the oxygen level.

For storage at 43% eRH^20^, forward selection with the E&R type model, starting with *TEMP* and log*OXY* only, showed a significant interaction (*P* < 0.001) and a quadratic *TEMP* effect (*P* = 0.004) provided by Model 2 (Table 2). The estimated *p*_50_ values for the different models (Table S3) reveal that Model 2 suffices. Because of the interaction, the shelf life extension factor for oxygen depends on the temperature. Halving the oxygen levels at 5°C gives an estimated extension factor of 0.5^−(1.12−0.0184×5)^ = 2.03 (±0.067), while at 13, 20 or 30°C, the extension factors equal 1.84 (±0.038), 1.68 (±0.035) and 1.48 (±0.054), respectively (Data S3). Because of the quadratic temperature effect and the interaction with oxygen in Model 2, the extension factor for dropping the temperature by 5°C depends on the temperature itself and on the oxygen level (Table S4). The temperature effect on shelf life was stronger with decreasing oxygen levels and with higher temperatures. According to Model 2, when at 5°C the oxygen level is reduced from 21% in the air to 5.2%, shelf life extends by a factor of 4.2 (±0.28), while at 13 and 20°C, this factor is respectively 3.4 (±0.14) and 2.8 (±0.12) (Data S3).

For storage at 60% eRH^20^, the forward selection with the E&R type model, starting with *TEMP* and *OXY* only, showed that the three additional terms (*TEMP* × log*OXY*, log*OXY* ^2^ and *TEMP* ^2^) are very significant (Table 2). There are differences between the *p*_50_ values for Models 0, 1, 2 and 3 and also with the *p*_50_ values for the Separate model (Table S5). Model 3, including the three very significant additional terms, can be used for practical purposes (Table 3). The estimate of the shelf life extension factor by reducing the oxygen level now depends on the oxygen level itself (due to the quadratic oxygen effect) and on the temperature (due to the interaction between temperature and oxygen in the model) (Table S6). According to Model 3, halving at 20°C, the oxygen level from 21 to 10.5%, from 10 to 5% or from 5.2 to 2.6% extends shelf life by respectively the factors 1.21 (±0.022), 1.03 (±0.036), or 0.89 (±0.046) (Data S3). The latter factor indicates a shortening of shelf life by a further reduction of oxygen at its lower levels, which was not observed in practice (Figures 1d and 2d). The shelf extension factor by reducing the temperature in Model 3 also depends on the temperature itself due to the quadratic temperature effect (Table S2), with an increase in shelf life extension at lower oxygen levels and also at lower temperatures. For example, for seeds stored in air at 60% eRH^20^ Model 3 provides a shelf life extension factor of 1.9 and 2.1 when the storage temperature is reduced from 20 to 15°C and from 13 to 8°C, respectively. This is in accordance with the slightly convex 60% eRH^20^ curves for *p*_50_ (Figure S3).

### A general model combining target eRH^20^ levels 16, 33 and 43%

For storage at 60% eRH^20^, an extended model with multiple selected terms seemed required. For the other target RH^20^ values the simple E&R type model, that is, with TEMP and log*OXY* only, seemed appropriate for practical purposes. Therefore, a combined model for levels 16, 33 and 43%, that is, excluding the 60% RH^20^ level, was fitted to the data. Forward selection starting with the E&R type model with *RH*, *TEMP* and log*OXY* only reveals that only the quadratic *RH* term and the interaction between log*OXY* and *RH* are significant (Tables S8 and S9). Parameter estimates and shelf life extension factors are given in Data S3. The extension factors are 1.72 for a temperature drop by 5°C, and when halving the oxygen level between 1.64 and 1.80, depending on the eRH^20^ level.

This modelling was repeated with the RH adjusted for the temperature effect, using Cromarty's equation (RH‐Cromarty) instead of eRH^20^ (Table S10). This revealed that the deviance of the starting model is larger than without adjustment (Table S8), indicating a worse fit. Also, the full model has a worse fit. From a modelling point of view, using RH‐Cromarty instead of eRH^20^ target does not seem critical.

### Summary

Table 4 summarises the effect of oxygen and temperature on the shelf life of the primed celery seeds using the simplest well‐fitting models per eRH^20^ level. For the three driest conditions, the combined analysis provides for halving the oxygen level, an average shelf life extension with a factor 1.72. Halving it twice from for example 21 to 5.2%, resulted in a 1.72^2^ = 3.0 times longer shelf life, and similarly decreasing the oxygen to 1% will result in a 1.72^4.39^ = 10.8 times longer shelf life. Reducing the storage temperature, under these three dry storage conditions, extended shelf life on average with a comparable factor of 1.72 for every 5°C decline in temperature. At the 60% eRH^20^ conditions, the situation clearly deviates with hardly or no effect of reducing the oxygen level, whereas reducing the temperature still contributed to extending the shelf life with an on average slightly stronger effect of cooler storage.

**Table 4:** Summary of the multiplicative effects of oxygen and temperature on the shelf life of primed celery seeds according to the simplest well‐fitting models. Selected models 0/1/2/3 are given in Table 2 and model *C*_2_ is the selected model for the combined target eRH^20^ levels 16, 33 and 43%, where eRH^20^ is the RH in the storage jars after equilibration at 20°C

eRH^20^	Selected model	Temperature			
		5°C	13°C	20°C	30°C
Shelf life extension factor by halving the oxygen levels					
16%	0	1.63			
33%	1	1.75			
43%	2	2.03	1.84	1.68	1.48
60%	3	1.05–2.02^a^	0.96–1.85^a^	0.89–1.72^a^	0.80–1.54^a^
16–43%	*C*_2_	1.64–1.80^b^			
Shelf life extension factor by dropping temperature by 5°C					
16%	0	1.75			
33%	1	1.92	1.77	1.65	1.48
43%	2	1.72–1.31^a^	1.90–1.45^a^	2.07–1.58^a^	2.34–1.79^a^
60%	3	2.56–2.03^a^	2.32–1.84^a^	2.13–1.69^a^	1.88–1.49^a^
16–43%	*C*_2_	1.72			

^a^ Range of oxygen levels from 5.2 to 99%.

^b^ Range for target eRH^20^ levels 16, 33 and 43%.

## DISCUSSION

### The quantitative oxygen effect on seed shelf life

A shelf life‐extending effect by storing seeds under reduced oxygen levels has been shown for several crops (Ellis & Hong, 2007; Groot et al., 2015; Harrison, 1966; Pérez‐García et al., 2007; Schwember & Bradford, 2011), while in some other studies, this effect was not observed (Morscher et al., 2015; Rafael Agostinho et al., 2021). Also in our experiments, we observed strong shelf life‐extending effects when the seeds were stored rather dry at 16, 33 and 43% eRH^20^ under hypoxia conditions.

Modelling the quantitative effect of oxygen level on the ageing of primed celery seeds showed that under the three driest conditions (16, 33 and 43% eRH^20^) a log–log linear relation was observed between the oxygen level and the rate of seed ageing (*p*_50_). The effect was rather constant between 5.2 and 99% oxygen. Modelling the oxygen effect per humidity condition, showed that at 16 and 33% eRH^20^, this log–log linear relation was independent of the temperatures used in our experiment (5 till 30°C), with shelf life extending factors of 1.63 and 1.75 for each halving of the oxygen level. At 43% eRH^20^, this factor varied with the temperature from 1.48 to 2.04. At 60% eRH^20^, there was hardly an extension of shelf life when oxygen levels were reduced from normoxia (21%) to lower levels.

Modelling the combined three driest conditions indicated that halving the oxygen level during storage increased the shelf life of the primed celery seeds by a factor of 1.72, in the same order of magnitude as indicated by the models for the separate eRH^20^ condition. The model implies an around 11 (1.72^4.4^) times longer shelf life at 1% oxygen as compared with normoxia. A further reduction, by creating anoxia conditions, will likely extend the shelf life of primed celery seeds even more. We were not able to create full anoxia conditions by flushing with nitrogen gas, but such anoxia conditions can relatively easily be obtained using iron powder based oxygen absorbers (Groot et al., 2015; Wu et al., 2021), as commonly used by the food industry (Cichello, 2015).

### The effect of varying oxygen levels at a higher humidity on seed shelf life

Under the more humid conditions at 60% eRH^20^, seed deterioration was even faster compared with the lower eRH^20^ levels, but the effects of decreasing the oxygen levels below 21% was much weaker (at 5, 13, or 20°C) or not measured (at 30°C). This diminishing effect at higher eRH^20^ and higher temperature is in agreement with the conclusions by Gerna et al. (2022) that the transition of the cytoplasm from a glassy to a rubbery and fluid state removes the advantage of storage at low oxygen levels. In their experiments, *P. densiflora* seeds stored at 45°C with a glassy cytoplasm (at 11 and 30% RH) showed a delayed deterioration under hypoxia (0.4% oxygen) compared with the storage under normoxia (21% oxygen), while there was no difference in the rate of deterioration between hypoxia or normoxia in seeds stored with a fluid cytoplasm (at 60 and 80% RH).

With sunflower seed embryos stored at 75% RH and 40°C under either ambient (21%) or high (75%) oxygen levels, deterioration was accompanied by a shift in the cellular redox state towards strong oxidising conditions, while no difference in the rate of viability loss was observed between the two oxygen levels (Morscher et al., 2015).

In our experiment upon storage at 60% eRH^20^, these effects of varying oxygen levels were also reduced with an increase in storage temperature. The diminishing oxygen effect with the increasing temperature would be compatible with an increase in the cytoplasmic molecular mobility in response to increases of both moisture level and temperature.

In our experiments, the glassy state of the celery seeds has not been determined, neither the seed moisture content. The estimated mean oil content of celery seeds equals 29% (Society for Ecological Restoration et al., 2023). Using Cromarty's equation, such an oil content gives a moisture content on dry weight basis of 4.5, 6.7, 7.8 and 9.7% at respectively an eRH^20^ of 16, 33, 43 and 60%. According to the curve fitted with data from soybean, common bean, pea and maize seeds, the average glass phase transition at 20°C occurs at around 10% moisture content on dry weight basis (Buitink & Leprince, 2004). When our estimations are correct, this means that for celery seeds stored at 16, 33 or 43 eRH^20^, the cytoplasm would be in the glassy state with a very low molecular mobility, while seeds stored at 60% and 20°C would likely be close to or at the onset of the glass transitions, which lead to an increase in molecular mobility. A shift towards a higher molecular mobility is increased by raising the storage temperature. Indeed, the effects of the oxygen levels are diminishing more strongly at higher temperatures (Figure 5d).

The shift from a glassy state towards increased molecular mobility may allow the onset of enzymatic ROS scavenging activity, which will increase with the molecular mobility of the cytoplasm and is enhanced by both an increase in seed moisture levels and storage temperature. When this hypothesis is correct, the enzymatic ROS scavenging may largely counteract deleterious oxygen effects up to 21%, but may be more limited at higher oxygen levels. For the latter, a further increase in the cytoplasmic molecular mobility would be needed to allow more enzyme activity, as observed in *P. densiflora* seeds stored at 45°C and 60 or 80% RH (Gerna et al., 2022) or sunflower embryos' stored at 40°C and 75% (Morscher et al., 2015). Detailed experiments are needed to analyse transitions in cytoplasmic molecular mobility in relation to the storage temperature, eRH or moisture content, for celery and other crops and to determine if there is a general or species‐specific glass transition temperature. To this end, the seed oil content and moisture sorption isotherms should also be taken into account (Hay et al., 2022).

### Deterioration induced by oxygen

Storage under hypoxia or anoxia conditions is frequently used to extend the shelf life of food (Cichello, 2015). Despite the role of oxygen in lipid oxidation and the advantages of hypoxia packaging, relatively little is known about the quantitative effect of the oxygen level on food quality deterioration (Johnson & Decker, 2015). It has been observed that reducing the oxygen level in the headspace from 2.0 to 1.0% decreased oxidation of a linoleate emulsion to a greater extent than a reduction from 21 to 2.0% oxygen (Marcuse & Fredriksson, 1968). Oxidation of high‐oleic safflower frying oil, placed in an Erlenmeyer flask and heated by blowing in a mixture of air and nitrogen gas at 180°C for 30 h, was completely inhibited at 2% headspace oxygen levels in contrast to 20% oxygen (Fujisaki et al., 2000). The relatively stronger effects on oil oxidation are supposed to be due to diffusion‐limited reaction rates, occurring only when oxygen levels are very low (Johnson & Decker, 2015).

With seeds, the increased oxygen levels likely result in enhanced ROS production and subsequent oxidation of DNA, RNA, proteins and lipids. With dry storage of wheat seeds, the viability loss is largely related to lipid oxidation (Wiebach et al., 2020). In the embryo, the main deteriorating effect induced by oxidation under dry storage conditions is most likely peroxidation of the unsaturated lipids in the cell and organellar membranes. For the maintenance of seed viability, the integrity and fluidity of these membranes are essential. Oxidation of unsaturated lipids in the cell membranes will enhance the risk of leakage, especially during imbibition. The latter explains the higher electro‐conductivity values for aged seeds, observed with the electro‐conductivity test for analysing Brassica seed vigour (ISTA, 2022; Matthews & Powell, 2006). Mitochondrial membranes are rather sensitive to ageing during seed storage resulting in loss of integrity (Benamar et al., 2003). In stored cabbage seeds, already after 3 months on the lab bench, anaerobic respiration is increased compared with that of fresh seeds (Kodde et al., 2011). Energy production by the mitochondria is essential for all germination processes, including damage repair (Bewley & Black, 2013; Małecka et al., 2021). Anaerobic respiration is a clear indication of less functional mitochondria. A likely reason may be the peroxidation of unsaturated mitochondrial membrane lipids, which reduces the membrane fluidity and integrity and subsequently disrupts the proton gradient over the inner mitochondrial membrane, hampering efficient aerobic respiration.

In seeds, tocopherols are the main non‐enzymatic and lipophilic antioxidants to protect the membranes. Previously, we showed that an increase in the oxygen level by storing dry cabbage seeds at 35% RH, under an elevated partial pressure of oxygen (EPPO) resulted in a faster decline in tocopherol levels and seed viability compared with storage under ambient oxygen levels (Groot et al., 2012). Under dry EPPO conditions, the decline in tocopherol and seed viability were linearly related, while such a decline in tocopherol levels was not observed in cabbage seeds stored at 85% RH.

### Consequences for seed ageing experiments and estimating seed longevity

The ‘improved’ E&R model for estimating seed longevity (Ellis & Roberts, 1980) has been applied to a considerable number of crops to estimate the species‐specific viability constants and the rate of seed ageing in relation to the storage temperature and seed moisture content. Although the Ellis & Roberts model gives a good indication of relative shelf life when comparing distinct species, the estimated absolute rate or the decline in shelf life often deviates from those found in practice. In general, to determine the species‐specific constants, seed samples are brought to different moisture contents, packaged in hermetically sealed pouches, stored at different temperatures and sampled for viability after different time intervals. When hermetically packaged, the amount of included oxygen can vary. Moreover, with 10 g lettuce seeds hermetically stored for 450 days in a 74 mL jam jar, initially with fresh air, oxygen levels declined in a negative exponential way, from 21% to around 15% in 112 days, to 10% in 250 days and to slightly above 5% in 450 days (Groot et al., 2015). This decline of oxygen levels is caused by the conversion of the oxygen molecules to ROS and subsequent oxidative reaction with seed macromolecular components (Apel & Hirt, 2004). This rate of oxygen decline will vary depending on the temperature, seed moisture content or eRH (Shahidi & John, 2013) and oxygen level itself (Groot et al., 2015). Therefore, reproducibility in the oxygen level and thus the rate of decline will be a challenge with experimental seed ageing under hermetic conditions, as used in protocols to determine species‐specific viability constants (Ellis & Roberts, 1980a, 1980b; Kraak & Vos, 1987). This is in contrast with the practice of seed storage in commercial warehouses, where the seeds are most often stored in woven bags or containers made from oxygen‐permeable material like cloth, carton or paper, allowing contact to air with around 21% oxygen. Our present data show that the oxygen level can have a strong effect on the rate of seed ageing. That makes it essential to incorporate the oxygen level in modelling the shelf life of seeds.

The oxygen‐extended E&R model allows us to model seed longevity with a logarithmic oxygen effect and an interaction with the eRH (or seed moisture level) and temperature. Moreover, for the application of the oxygen‐extended E&R model, the molecular mobility of the cytoplasm should also be taken into account. Ageing was faster with increasing storage temperatures, with a near linear relation between the temperature and *p*_50_ on the log scale. The rate of decline in *p*_50_ in relation to an increasing storage temperature was rather comparable for the different oxygen levels at the two eRH^20^ levels of 15 and 33% (Figure S4A and 4B). Under these driest storage conditions, the quantitative oxygen effect was independent of the temperatures tested (5–30°C). At an eRH^20^ of 43%, the temperature effect on shelf life was stronger with decreasing oxygen levels and the effect of reducing the oxygen levels was relatively stronger at higher temperatures (Table 4; Figure S4C). A reason could be a stronger increase in water activity (eRH) upon storage at higher temperatures (Cromarty et al., 1982 [revised 1990]). Moreover, in biological systems, cellular viscosity still decreases above the glass transition temperature with a further rise in temperature (Buitink & Leprince, 2004). which will increase also the mobility of molecular oxygen and ROS. Another potential reason could be a temperature effect on the rate of oxygen diffusion through the water layer covering macro molecules, including the membrane lipids (Barden & Decker, 2016).

Modelling the temperature effect with the primed celery seeds stored at 16, 33 and 43% eRH^20^ showed that dropping the temperature by 5°C extended the shelf life by a factor between 1.48 and 1.92, which is in the same order of magnitude as from one of Harrington's rules of thumb (Harrington, 1972), which assumes a doubling of shelf life for each temperature drop of 5.6°C (10°F). The stronger ageing effect, observed at the relatively higher temperatures, will largely be due to accelerating the kinetics of all (bio)chemical reactions. It may also be related to the temperature influence on the water activity, although replacing eRH^20^ by eRH using Cromarty's equation (Cromarty et al., 1982 [revised 1990]) in our oxygen extended E&R model did not provide a better fit.

### Beyond primed celery seeds

To enable modelling the effect of reduced oxygen levels and their interaction with temperature and storage RH, supporting experimental data need to be acquired in a reasonable time frame. This requires seeds with a relative short shelf life, and we have chosen primed celery seeds. The question arises if the observed quantitative effect of hypoxia also holds for non‐primed seeds and seeds of other species. From food science, it is known that drying to very low moisture levels increases the sensitivity to oxidation (Labuza & Dugan, 1971). The theory behind this is that restriction of oxygen access to macromolecules through a monolayer of water or rather water saturation of the lipid polar groups, and removal of water under ultra‐dry conditions promotes lipid oxidation, including in the membranes (Barden & Decker, 2016). Seeds stored for 3 years ultra‐dry and in large volume exicators deteriorated faster compared with seeds stored at slightly higher seeds moisture content (Chai et al., 1998), confirming the results of food science studies. However, seeds stored hermetically ultra‐dry (eRH^20^ 10–12%) and with an enclosure of only small volumes of air showed no difference after 10 years of storage compared with the seeds stored at higher moisture levels (eRH^20^ 19–55%) (Hong et al., 2005). Another clear demonstration of large positive effects of hypoxia or anoxia on seed viability under ultra‐dry storage was demonstrated with 38–40 years of storage of *Brassicaceae* seeds (Pérez‐García et al., 2007). The authors had stored the seeds above regenerated silica gel in flame‐sealed glass tubes after replacing the atmosphere with carbon dioxide. Seeds with an initial high germination frequency had almost maintained their high quality.

Most publications on storage of seeds under different oxygen levels are comparing storage after flushing with air, 100% oxygen or 100% nitrogen gas (e.g. Roberts, 1961), making comparison with our model difficult, regardless of the scale used to express oxygen levels. Primed and non‐primed lettuce seeds have been stored at 2% oxygen or 21% oxygen at 32% RH and 37°C (Schwember & Bradford, 2011). In that experiment, analysis of the oxygen effect on the frequency of normal seedlings showed an around 20% extension of shelf life by storage at 2% compared with storage at 21% oxygen (for both primed and non‐primed seeds, as estimated from the figure 2 in that publication). The extension of shelf life was much smaller than what we observed with primed celery seeds. More experiments with various crops and wild plants are needed to obtain a broader picture.

Our storage experiments with primed celery seeds lasted 7 years, required to observe an ageing effect of storage at oxygen levels of around 1%. Most other seeds have a longer shelf life, and comparable storage experiments under dry conditions and reduced oxygen levels will take far longer than 7 years. As an alternative approach to analyse the quantitative effect of oxygen on seed ageing, we have initiated a storage experiment of non‐primed onion and lettuce seeds at 30°C and 30% RH, under different mixtures of oxygen and nitrogen gas at a high pressure of 20 MPa (200 bar) employing our EPPO experimental ageing method (Buijs et al., 2020; Groot et al., 2012). Due to the higher partial oxygen pressures, this EPPO experimental ageing method allows an acceleration of the ageing without the need for high temperatures or relative humid storage. The various oxygen levels were produced by using the same high pressure, but with different ratios of air, oxygen and nitrogen gas. Preliminary results indicate that the ageing of the lettuce and onion seeds also responded in a log–log linear relationship to the different oxygen levels, with for both species, a shelf live extension factor of around 1.8 by halving the oxygen levels (S.P.C. Groot & B. van Sleuwen, unpublished data). This is in the same order as observed with the primed celery seeds under ambient pressure (100 kPa) in the present study. This alternative experimental approach indicates that also for lettuce and onion oxygen has a strong quantitative log–log linear effect on seed longevity under dry storage conditions.

### Practical application

Our data confirmed that storing seeds under strongly reduced oxygen levels can extend their shelf life considerably and decrease economic losses due to seed ageing. Oxygen levels below 1% can be easily obtained by storing seeds hermetically under a near vacuum as done by several genebanks (van Treuren et al., 2013). Oxygen absorbers, which are frequently used by the food industry, can provide anoxia conditions (Cichello, 2015). When using commercial oxygen absorber packages, care should be taken that most of these contain also a moisturiser, for instance, moist vermiculite, to fasten oxygen depletion. When such oxygen absorbers are applied with a small number of seeds, the addition of a desiccant like a small amount of dry silica gel can avoid an increase in the eRH without hampering oxygen depletion (Wu et al., 2021).

For storage of larger seed volumes multilayered polymer big bags are available on the market. These bags have a very low oxygen and water vapour transmission rate and rather low oxygen levels can be achieved using air removal by creating a vacuum and flushing with nitrogen gas. Performing this twice, with a few hours interval, is rather effective (M.‐J. van Litsenburg, VacQPack, personal communication).

The relatively large effect of hypoxia storage does not replace the need for adequate drying of the seeds. Optimally, the seeds should be dried at least below their glass transition point, likely for most crops below 50% eRH^20^ (Buitink & Leprince, 2004; Gerna et al., 2022), preferably till 20–30% eRH^20^ and maybe even lower for genebank storage. Also it is still advantageous to store seeds under temperature‐controlled conditions. However, maintenance of cooled storage is rather costly and challenging under tropical conditions (Bradford et al., 2018). Moreover, temperature‐controlled storage is often not performed by dealers or farmers. Hermetic packaging in combination with nitrogen flushing, can have a very significant effect on the maintenance of seed vigour for a longer period and can provide farmers with on average higher quality seeds. Oxygen levels can also be decreased by flushing with carbon dioxide gas, as is occasionally done with food products, but this is not recommended for seeds. Rice seeds stored at 12% moisture content under carbon dioxide deteriorated faster compared with storage under nitrogen gas (Roberts, 1961). The negative effect of carbon dioxide is likely due to acidification of the water still present in the seeds, which may give rise to increased permeability of the membranes, while nitrogen gas is neutral in this respect (Mitcham et al., 2006).

For the conservation of genetic diversity in genebanks, long shelf life conditions are very important. Our results from storage at temperatures between 5 and 30°C indicate that the viability of orthodox seeds can be extended by the combination of low moisture, low temperature and low oxygen. Studies with food have shown that lipid oxidation reactions proceed at sub‐zero temperatures, although at a lower rate, and are the primary cause of quality loss in frozen foods (Calligaris et al., 2007). We therefore recommend implementing the advantage of hypoxia storage in an updated version for genebank standards (Food and Agriculture Organization of the United Nations, 2013).

## MATERIAL AND METHODS

### Seed material

Celery (*A. graveolens* L.) seeds were primed at Bejo Seeds (Warmenhuizen, The Netherlands) for 14 days with −1.2 MPa polyethylene glycol (PEG) 6000, in constant light, at 15°C according to a traditional priming protocol (van der Toorn & Karssen, 1992). After priming, the seeds were rinsed with water to remove the PEG and dried for 24 h with ambient air heated to 30°C and subsequently at 20°C and 30% RH.

### Storage conditions

After arrival at Wageningen University & Research, a small random portion of the seeds was used to obtain nine control samples to determine the initial viability in germination tests. The other seeds were packaged in individual paper sample bags, each containing approximately 0.2 g seeds (around 630 seeds) and stored in transparent 194 mL glass jars (Figure S3). For each of the 96 storage conditions, 10 samples were stored in a single jar. The jars also contained 40 g of RH buffering silica gel (Bartolotta et al., 1991; Groot et al., 2012) equilibrated at either one of the four target RH values (targets were 15, 30, 45 and 60%), using different ratios of dry silica gel mixed with silica gel humidified at 70% RH.

The jars were hermetically closed with a metal lid and plastisol sealing. A hole was made in the metal lid with a nail, to allow flushing with oxygen and/or nitrogen gas in mixtures of 0, 5.2, 10, 21 (air), 50 or 100% oxygen. After 2 min of flushing at a rate of 0.5 L min^−1^, the hole was closed with self‐adhesive aluminium tape (Griffon, Netherlands). The laboratory in Wageningen is situated at 8 m above sea level, making the average air pressure in the lab close to 100 kPa (1 bar). The oxygen levels in the jars were tested at least 20 min after closure using optical oxygen sensor dots (precision ±1%, PreSens—Precision Sensing GmbH, Regensburg, Germany) placed inside the glass wall of each jar. The sensor was calibrated with a sensor dots placed in fresh air (21% oxygen) or in a solution of 1 g L^−1^ sodium sulphite (Na_2_SO_3_) dissolved in water (0% oxygen).

Since 2 min of flushing with nitrogen gas did not remove all the original air, the lowest oxygen level was around 1%. Similarly, flushing with 100% oxygen resulted in an oxygen level of around 99%. The jars with samples were placed in darkness, in temperature‐controlled incubators at 5, 13, 20 or 30°C. Thus, 96 different storage conditions were employed by a combination of six oxygen levels (≈1, 5.2, 10, 21 [air], 50 and 99%), four storage temperatures (5, 13, 20 and 30°C) and the four target RH levels (15, 30, 45 and 60%). To retrieve a sample for a germination test, the jars were temporarily removed from the incubators and placed in the lab at 20°C for at least 2 h and the internal oxygen levels were measured using the oxygen sensor dots. The equilibration at 20°C was needed because temperature influences the fluorescence pattern from the sensor dots and thereby the measurement of the oxygen level. A few jars with deviating oxygen levels, likely due to leakage were discarded. As a consequence, the number of storage intervals decreased for those conditions. After retrieval of a seed sample, the jars were flushed again with the appropriate mixture of oxygen and nitrogen gas, closed and stored again at the target temperature.

The individual seed samples were too small for accurate analysis of their equilibrium RH (eRH) or moisture content. Therefore, the eRH of the buffering silica gel was determined at 20°C after retrieval of the last sample by measuring the water activity using an HC2‐AW probe connected to a HygroLab 3 display unit (Rotronic Measurement Solutions, Hauppauge, New York, USA), assuming the eRH (in %) is 100‐fold the measured water activity. These measurements showed actual average eRH values at 20°C of 16.0 ± 2.2% (mean ± standard deviation), 32.8 ± 1.0%, 43.4 ± 1.2% and 60.3 ± 1.6% respectively (Figure S4). This eRH measured at 20°C is referred to as eRH^20^. It should be noted that the storage temperature has a slight effect on the eRH, with lower levels at lower temperatures, and higher levels at higher temperatures (Reid, 2020). Since the eRH was determined at 20°C, a correction for storage at the other temperatures was calculated using Cromarty's equation (Cromarty et al., 1982 [revised 1990]) (Table 5).

**Table 5:** Mean measured equilibrium relative humidity (eRH) levels of the buffering silica gel at 20°C (eRH^20^), where eRH^20^ is the RH in the storage jars after equilibration at 20°C, and the theoretical eRH at different storage temperatures, calculated using the Cromarty's equation (Cromarty et al., 1982 [revised 1990])

Target eRH	Mean eRH measured at 20°C (eRH^20^)	Calculated eRH at 5°C	Calculated eRH at 13°C	Calculated eRH at 30°C
15%	16.0%	12.5%	14.3%	18.5%
30%	32.8%	26.2%	29.7%	37.3%
45%	43.4%	35.2%	39.6%	48.8%
60%	60.3%	50.6%	55.9%	66.2%

### Sampling and germination tests

Individual seed samples were retrieved from storage at various time intervals and seed survival was determined using a germination test on wet filter paper (Allpaper, Didam, The Netherlands) at 20°C without light (Figure S5). Around 100–200 seeds were used per germination test. Radicle protrusion of at least 1 mm was used as the criterion for survival. This was scored after 10 days for each seed by image analysis using the GERMINATOR system (Joosen et al., 2010). Intervals between successive germination tests were 1–4 weeks from the start of the storage experiment and around half a year near the end of the experiment. Jars exposed to relative higher temperature, humidity and oxygen levels, were sampled more frequently during the first year of the storage experiment. The last germination test, with samples stored at 1% oxygen and 16 or 32.8% eRH^20^, was performed after 2632 days (7.2 years). At that moment, samples from the other treatments were already depleted. The initial viability of the seed lot was estimated by averaging the germination proportion of the nine control samples. Two out of the 773 germination tests were considered non‐reliable, as they indicate a considerable increase in the proportion of germinating seeds after a clear decline at the previous sampling intervals, and these were excluded from the data.

### Modelling seed ageing

The observed germination data, that is, the number of germinated seeds with respect to the total number of seeds per test, were modelled using a generalised linear model (GLM) with the binomial distribution and a probit link, similar to the Ellis & Roberts (E&R) viability model (Ellis & Roberts, 1980; Hay et al., 2014). Overdispersion with respect to the binomial distribution was accounted for by inflating the binomial variance with an overdispersion factor, and this factor was estimated by the mean deviance of the fitted model (McCullagh & Nelder, 1989). The E&R viability model can be written as $\nu =K_{i}-p/\sigma_{S}$, in which ν denotes the probit of the viability, $K_{i}$ is the probit of the initial viability at zero experimental storage days, p is the storage time in days, and $\sigma_{S}$ is the time for viability to fall by one probit, which depends on the storage conditions S. The purpose of the modelling was to find a good fitting explicit mathematical relationship between $\sigma_{S}$ and the storage conditions. The estimated storage time when the seed lot viability has declined to 50%, further denoted by *p*_50_, is given by $K_{i}\sigma_{S}$, which follows from the fact that probit (50%) = 0. The parameter $K_{i}$ was set to the fixed value 1.61 = probit (94.6%), based on the mean germination of the nine control samples weighted by the number of seeds in each control sample.

In an initial statistical analysis, a probit model was fitted with a different slope $\sigma_{S}$ for each of the 96 combinations of temperature, oxygen and eRH^20^. This model is a simple GLM in which $K_{i}$ is a so‐called offset. The $\sigma_{S}$ parameters were estimated using the glm() function in R statistical software (R Core Team, 2023) with the quasi‐binomial distribution and a probit link. Graphs revealed that the corresponding log_10_(*p*_50_) values per eRH^20^ level were approximately linearly related to both Temperature (*TEMP*) and log_10_(Oxygen), further denoted by logOXY. This implies, per eRH^20^ level, a linear model log_10_(*p*_50_) ≈ *D*_0_ − *C*_1_ *TEMP* − *C*_2_ log*OXY* in which *D*_0_, *C*_1_ and *C*_2_ are parameters. Since *p*_50_ = $K_{i}\sigma_{S}$, it follows that a more concise model per eRH^20^ level is given by $\sigma_{S}=10^{C_{0}-C_{1}\text{TEMP}-C_{2}\log\mathrm{OXY}}$ with $C_{0}=D_{0}-\log_{10}\left(K_{i}\right)$. The model per eRH^20^ level then reads.

(1) $$\text{probit}\;\left(\nu \right)=K_{i}-p/10^{C_{0}-C_{1}\text{TEMP}-C_{2}\log\mathrm{OXY}}$$

This is similar to the E&R model (Ellis & Roberts, 1980; Hay et al., 2003) with an extension for the logarithmic oxygen effect and exclusion of the moisture content effect. An extension to the model in Equation (1), including a humidity effect, quadratic effects and interactions is given in Appendix S4. The model in Equation (1) only has three parameters, instead of 24 $\sigma_{S}$ parameters for each eRH^20^ level. The interpretation of the parameters $C_{1}$ and $C_{2}$ is best given in terms of the shelf life extension factor, see below. Note that the power 10 function ensures that $\sigma_{S}$ is positive giving declining viability curves in time. This is a non‐standard GLM, which was fitted using the optim() function in R. To increase numerical precision all storage terms in the fitted model were standardised to have mean 0 and variance 1. Forward selection, employing a quasi‐deviance test, was used to see whether quadratic terms in Temperature or log*OXY* or their interaction gave an improved fit.

Additionally, a combined model for target eRH^20^ levels of 16, 33 and 43%, that is excluding the 60% level, was developed. It should be noted that whereas the original E&R viability model (Ellis & Roberts, 1980; Hay et al., 2014) describes the effect of humidity using the moisture content of the stored seed sample, we used in our experimental modelling the eRH at 20°C (eRH^20^), which equals the water activity of a sample stored at that temperature.

Using the models, it can be calculated with what multiplicative factor the $p_{50}$ value is extended when for instance the oxygen level is halved or the storage temperature is decreased by 5°C. Standard errors of these multiplicative factor were obtained by the delta method (Oehlert, 1992). The formula for this so‐called ‘shelf life extension factor’ is given in Appendix S4. For the model given in Equation (1), each drop of 1°C extends the shelf life with a factor of $10^{-C_{1}}$, while for each halving of the oxygen level, the extension factor equals $0.5^{-C_{2}}$. Appendix S5 contains the R code and associated output for all the fitted models.

## AUTHOR CONTRIBUTIONS

*Conceptualization*: SPCG and PWG; *investigation*: SPCG, PWG, DSV and JK; *writing—original draft*: SG and PWG; *writing—review and editing*: SPCG. PWG, DSV and JK; *funding acquisition*: SPCG and DSV.

## CONFLICT OF INTEREST

The authors declare no conflict of interest.

## Supporting information


**Data S1.** Experimental data.

**Supplementary S2.** Figures and Tables.

**Data S3.** Estimated shelf life extension factors.

**Appendix S4.** Formula for the shelf life extension factor for the E&R model with several covariates.

**Appendix S5.** R scripts used in the modelling.

## Data Availability

The original data and R‐scripts used in the modelling are available in the supplementary material of this article.
