# Supplementary material for: Modelling the quantitative effect of oxygen on the ageing of primed celery seeds
Source: Plant J. 2025 Apr 17;122(1):e70066. doi: 10.1111/tpj.70066 (PMC12005833; doi:10.1111/tpj.70066)
Supplement: Supplementary file 2 — Supplementary S2. Figures and Tables. [file TPJ-122-0-s004.pdf]

Supporting Information 01 (Figures and Tables)

Modelling the quantitative effect of oxygen on the ageing of primed celery seeds

Steven P.C. Groot[a, b]*, Paul W. Goedhart[a], Deborah de Souza Vidigal[c] & Jan Kodde[a]

[a] Wageningen University & Research, Wageningen, The Netherlands.

[b] International Seed Academy, Didam, The Netherlands

[c] Bejo Zaden B.V., Warmenhuizen, The Netherlands.

*e-mail corresponding author: steven@seedacademy.org

Contents

Figure S1. Storage duration when seed lot viability had declined to 50% (p_{50}) as a function of the oxygen level.

Figure S2. Storage duration when seed lot viability had declined to 50% (p_{50}) as a function of the temperature.

Figure S3. The seed storage system that was used in the experiment.

Figure S4. Individual eRH^{20} measurements at 20°C per target RH.

Figure S5. Germination test with primed celery seeds stored under different environmental conditions.

Table S1. Estimated p_{50} values for the different models for 16% eRH^{20}.

Table S2. Estimated p_{50} values for the different models for 33% eRH^{20}.

Table S3. Estimated p_{50} values for the different models for 43% eRH^{20}.

Table S4. Estimated shelf life extension factors for reducing temperature for Model 2 at 43% eRH^{20}.

Table S5. Estimated p_{50} values for the different models for 60% eRH^{20}.

Table S6. Estimated shelf life extension factors for halving the oxygen level for Model 2 at 60% eRH^{20}.

Table S7. Estimated shelf life extension factors for reducing temperature for Model 2 at 60% eRH^{20}.

Table S8. Analysis of deviance for the combined E&R model for eRH^{20} levels 16, 33 and 43%.

Table S9. Estimated p_{50} values for the different models for the combined E&R model for eRH^{20} levels 16, 33 and 43%.

Table S10. Analysis of deviance for the combined E&R model employing RH according to the Cromarty equation.

Figure S1.

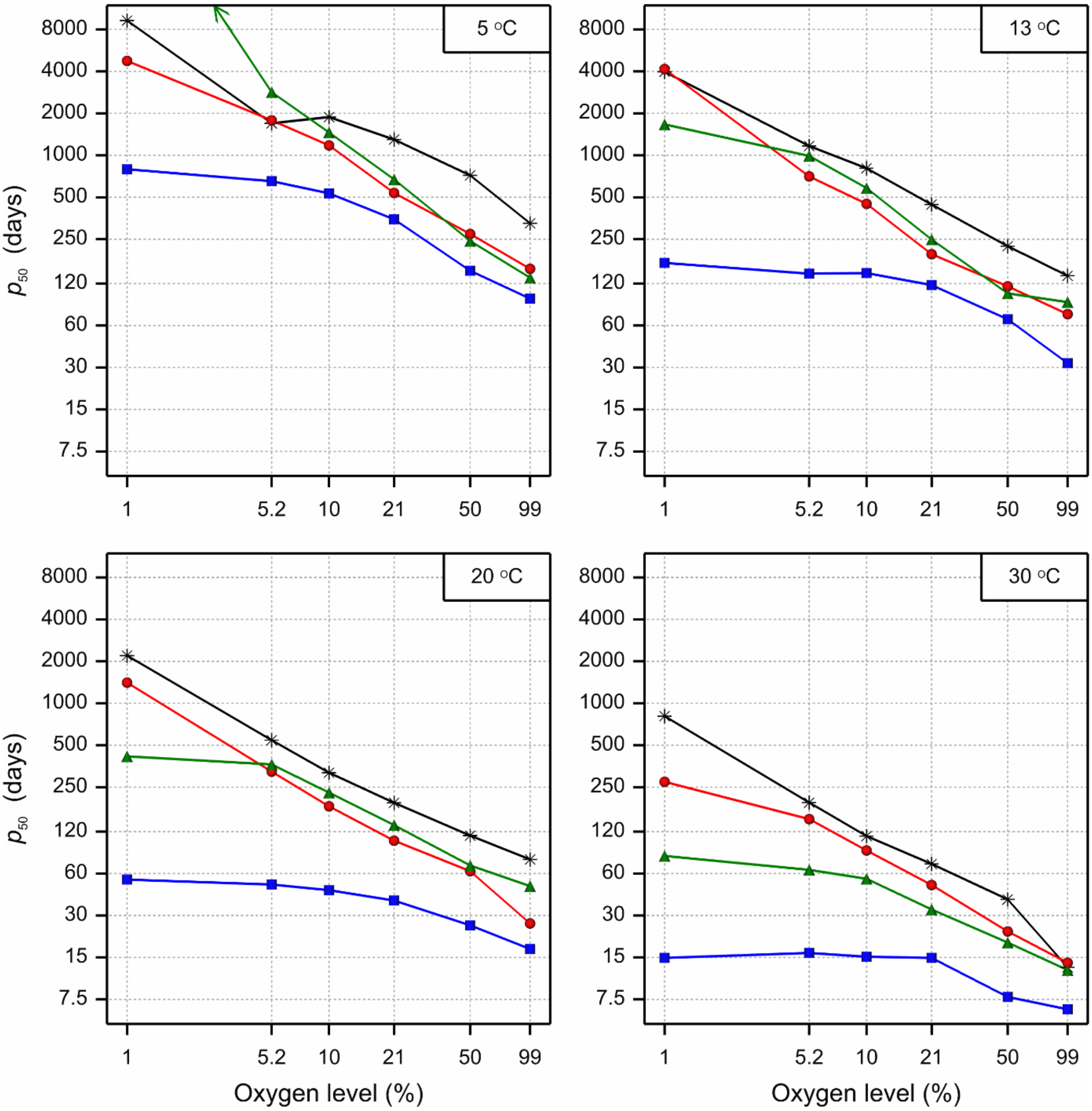

Figure S1. Storage duration when seed lot viability had declined to 50% (p_{50}) on a log scale as a function of the oxygen level (on a volume basis) on the log scale. The RH in the storage jars was measured after equilibration at 20°C (eRH^{20}). The actual eRH during storage will have deviated slightly, see Table 1.The four panels represent the four temperature levels, each with separate curves for the different eRH^{20} levels (✱ 16%, ● 33%, ▲ 43%, ■ 60%). The position of the '1%' oxygen concentration is less accurate compared with that of the other concentrations, because of the log-scale and the relative higher uncertainty of the actual oxygen concentration.

Figure S2.

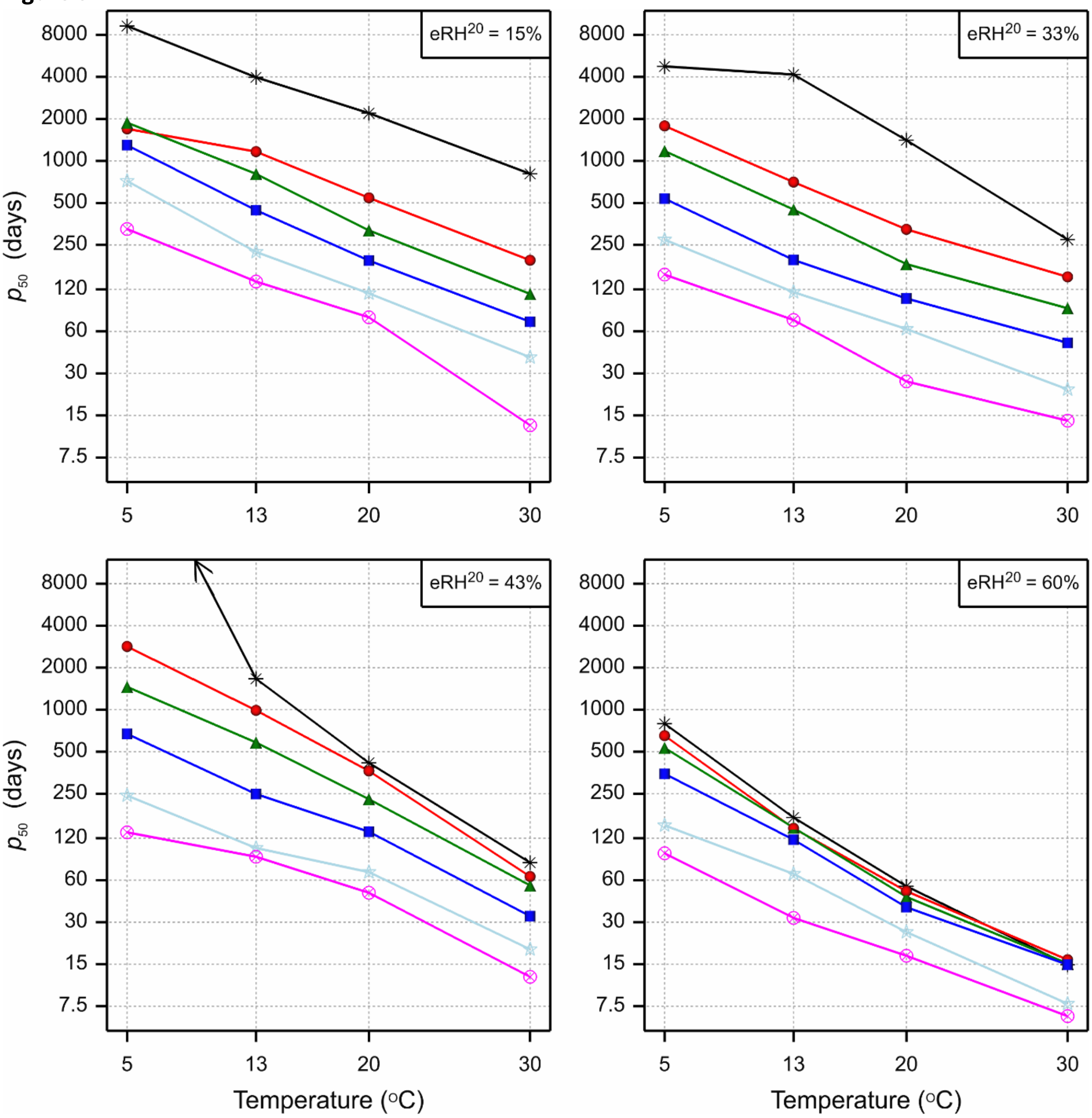

Figure S2. Storage duration when seed lot viability had declined to 50% (p_{50}) on a log scale as a function of the temperature. The RH in the storage jars was measured after equilibration at 20°C (eRH^{20}). The actual eRH during storage will have deviated slightly, see Table 1. The four panels represent the four eRH^{20} levels, each with separate curves for the different oxygen levels (✱ 1%, ● 5.2%, ▲ 10%, ■ 21%, ✡ 50% and ⊗ 99%).

Figure S3.

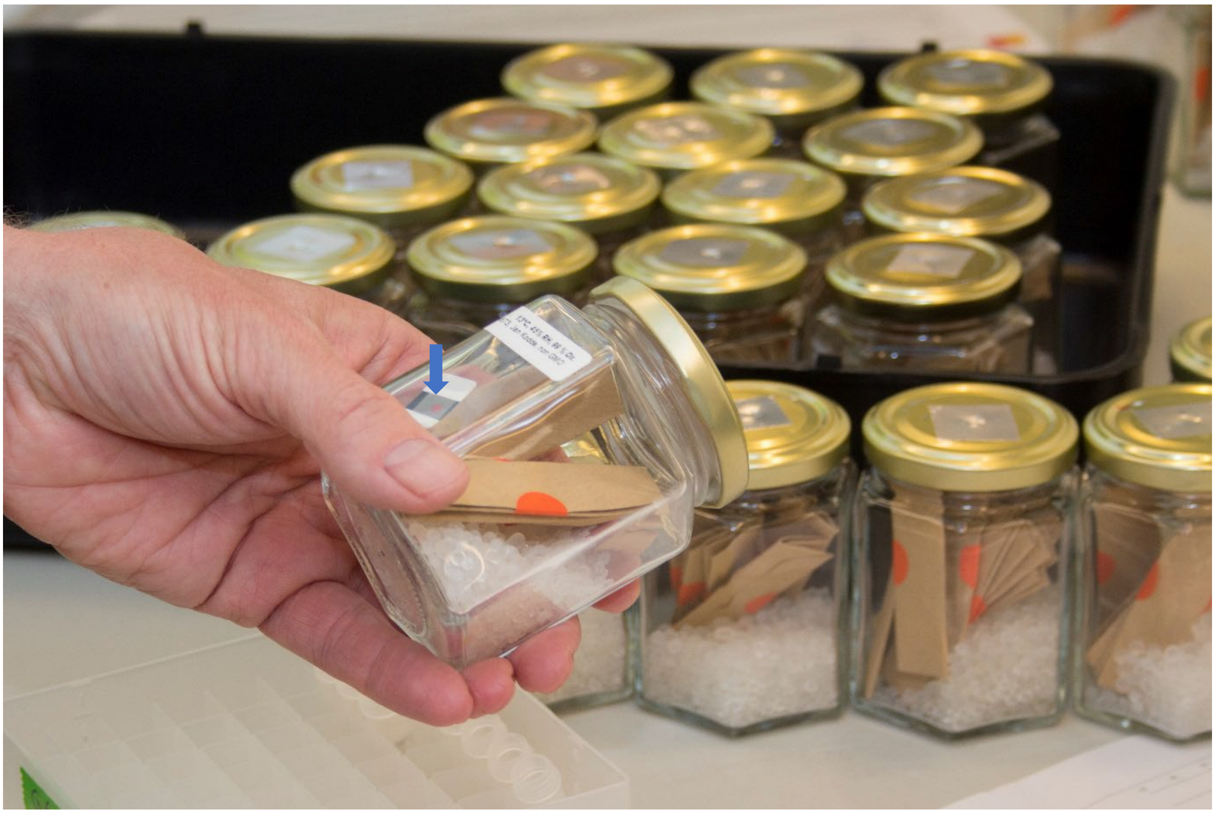

Figure S3. The seed storage system that was used in the experiment. Seed samples were packed in folded paper bags and stored in hermetic sealed transparent glass jars. Silica gel with an estimated equilibrium relative humidity of 15, 30, 45 or 60% was added to the jar to buffer the RH upon flushing with the various gas mixtures. A hole in the lid, used for flushing, was afterwards sealed with self-adhesive aluminium tape. The oxygen levels in the jars were tested at least twenty minutes after closure through the pink optical oxygen sensor dot (below the blue arrow) placed inside each jar against the glass. The actual equilibrium relative humidity at 20 °C (eRH^{20}), provided by the buffering silica gel, was measured after retrieval of the last sample and turned out to be on average 15, 33, 43 or 60% .

Figure S4.

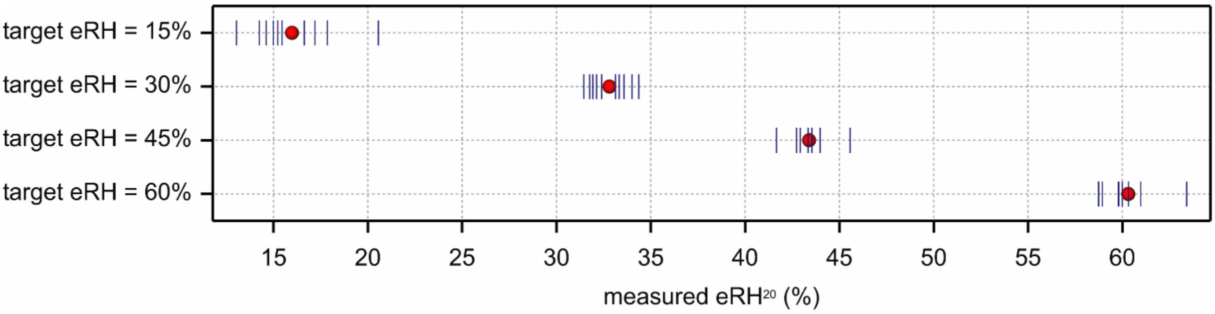

Figure S4. Individual equilibrium RH measurements at 20°C per target RH. The red dot is the mean of these measurements and is referred to as eRH^{20}. The data were obtained by analysing the water activity of the buffering silica gel at 20°C after retrieval of the last sample from individual jars stored under a range of conditions.

Figure S5.

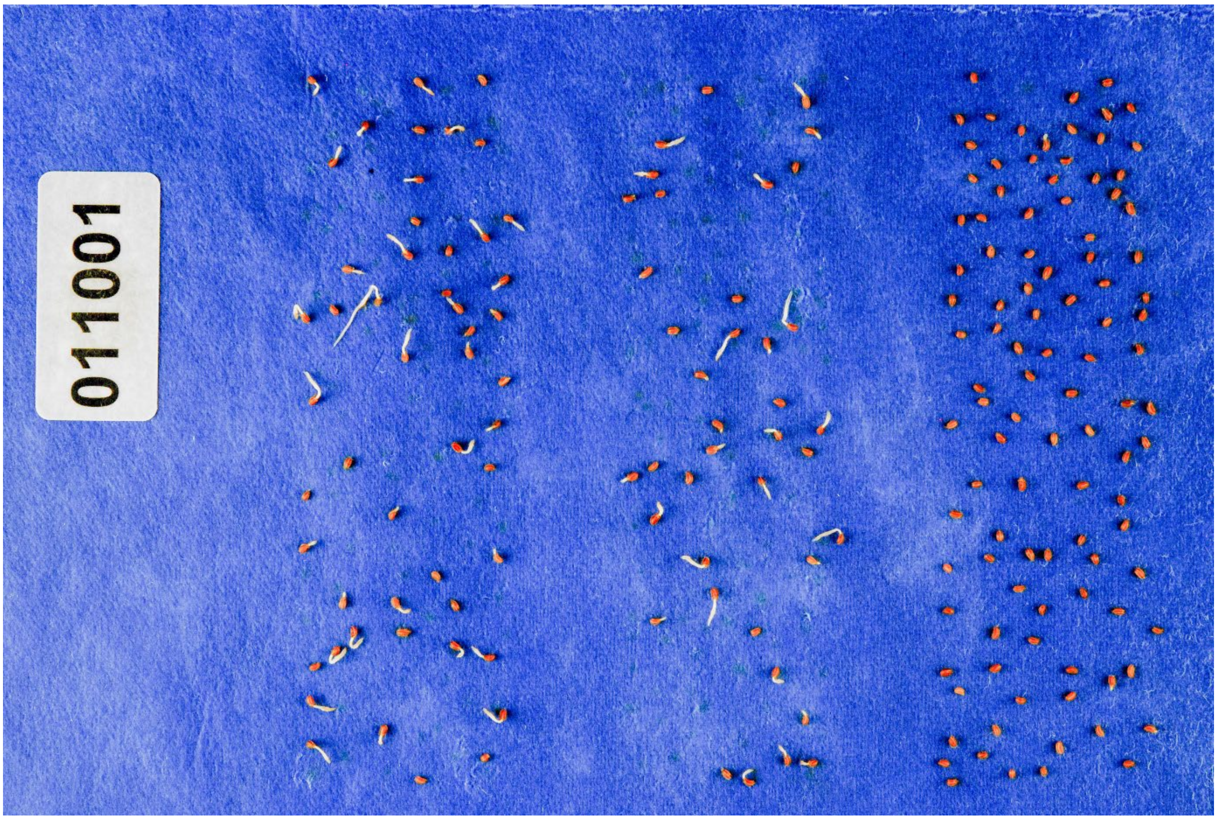

Figure S5. Germination test with primed celery seeds stored under different environmental conditions. Approximately 100-200 seeds were placed on a bleu blotter, imbibed and incubated at 20 °C in the dark. At five and ten days after imbibition, a picture was taken. On day five germinated seeds, showing root protrusion of at least 1 mm, were counted and removed to avoid disturbance in the image analyses at day ten by elongated roots. This picture was made ten days after imbibition. The frequency of germinated seeds was determined by image analysis using the GERMINATOR system. The sticker on the left contains a digit code for identification of the tray by image analysis.

Table S1. Estimated p_{50} values in days for the different models for 16% eRH^{20}.
Values are according to a separate fitted curve for each storage combination, Model 0 for the E&R model with temperature *TEMP* and log*OXY* (= log(oxygen %)) only, and for Models 1, 2 and 3 the terms *TEMP* x log*OXY,* log*OXY*^{2} and *TEMP*^{2} are added according to the forward selection. The RH in the storage jars was measured after equilibration at 20°C (eRH^{20}). The actual eRH during storage will have deviated slightly, see Table 1.

TEMP (°C)	*OXY* (%)	*Separate*	*Start* *Model 0*	+ *TEMP* x log*OXY* *Model 1*	+ log*OXY*^{2} *Model 2*	+ *TEMP*^{2} *Model 3*
5	5.2	1697	2967	2638	2452	2431
5	10	1880	1868	1749	1758	1747
5	21	1297	1105	1097	1146	1141
5	50	718	598	636	648	646
5	99	325	369	414	393	391
13	5.2	1165	1207	1142	1074	1075
13	10	807	760	735	745	747
13	21	443	449	446	467	469
13	50	223	243	248	253	254
13	99	137	150	157	148	148
20	5.2	544	549	549	522	524
20	10	318	346	344	351	353
20	21	193	205	203	213	214
20	50	112	111	109	111	111
20	99	76	68	67	63	63
30	5.2	194	178	193	186	186
30	10	111	112	116	120	120
30	21	70	66	66	69	69
30	50	39	36	34	34	34
30	99	13	22	20	19	18

Table S2. Estimated p_{50} values in days for the different models for 33% eRH[20].
Values are according to a separate fitted curve for each storage combination, Model 0 for the E&R model with temperature *TEMP* and log*OXY* (= log(% oxygen)) only, and for Models 1, 2 and 3 the terms $TEMP^2$, *TEMP* x log*OXY* and $\log OXY^2$ are added according to the forward selection. The RH in the storage jars was measured after equilibration at 20°C (eRH[20]). The actual eRH during storage will have deviated slightly, see Table 1.

TEMP (°C)	*OXY* (%)	*Separate*	*Start* *Model 0*	+ $TEMP^2$ *Model 1*	+ *TEMP* x log*OXY* *Model 2*	+ logOXY^2 *Model 3*
5	5.2	1782	1649	1761	1776	1826
5	10	1177	968	1039	1035	1047
5	21	536	529	572	566	565
5	50	272	261	284	283	280
5	99	153	149	164	165	163
13	5.2	705	745	690	696	701
13	10	448	437	407	406	405
13	21	195	239	224	222	221
13	50	115	118	111	111	110
13	99	72	68	64	65	65
20	5.2	323	372	339	342	340
20	10	182	218	200	199	198
20	21	103	119	110	109	109
20	50	62	59	55	54	55
20	99	26	34	31	32	32
30	5.2	148	138	146	147	145
30	10	88	81	86	86	86
30	21	50	44	47	47	47
30	50	23	22	24	23	24
30	99	14	12	14	14	15

Table S3. Estimated p_{50} values in days for the different models for eRH^{20} 43%.
Values are according to a separate fitted curve for each storage combination, Model 0 for the E&R model with temperature *TEMP* and log*OXY* (= log(% oxygen)) only, and for Models 1, 2 and 3 the terms *TEMP* x log*OXY*, $TEMP^2$ and $logOXY^2$ are added according to the forward selection. %. The RH in the storage jars was measured after equilibration at 20°C (eRH^{20}). The actual eRH during storage will have deviated slightly, see Table 1.

TEMP (°C)	*OXY* (%)	*Separate*	*Start* *Model 0*	+ *TEMP* x log*OXY* *Model 1*	+ $TEMP^2$ *Model 2*	+ $logOXY^2$ *Model 3*
5	5.2	2836	2190	3101	2700	2825
5	10	1458	1282	1567	1382	1389
5	21	671	698	722	646	634
5	50	243	343	292	266	261
5	99	132	196	143	132	133
13	5.2	989	844	951	996	1032
13	10	582	494	532	561	560
13	21	249	269	275	293	287
13	50	102	132	127	137	135
13	99	88	76	69	75	76
20	5.2	364	366	338	366	376
20	10	229	214	207	224	223
20	21	134	117	118	129	126
20	50	69	57	62	67	66
20	99	49	33	37	40	41
30	5.2	64	111	77	71	72
30	10	55	65	54	49	49
30	21	33	35	35	32	32
30	50	19	17	22	20	20
30	99	12	10	15	13	14

Table S4. Estimated shelf life extension factors (ratio + standard errors) at different oxygen levels after reducing the temperature by 5°C, starting at the indicated temperature, for Model 2 at eRH^{20} level 43%. The RH in the storage jars was measured after equilibration at 20°C (eRH^{20}). The actual eRH during storage will have deviated slightly, see Table 1.

Temperature (°C)	5 °C cooler	Oxygen (%)	log (oxygen)	ratio	s.e. ratio
5	0	99	2.00	1.31	0.082
5	0	50	1.70	1.40	0.086
5	0	21	1.32	1.51	0.095
5	0	10	1.00	1.62	0.107
5	0	5.2	0.72	1.72	0.122
13	8	99	2.00	1.45	0.054
13	8	50	1.70	1.54	0.051
13	8	21	1.32	1.67	0.054
13	8	10	1.00	1.79	0.066
13	8	5.2	0.72	1.90	0.083
20	15	99	2.00	1.58	0.047
20	15	50	1.70	1.68	0.036
20	15	21	1.32	1.82	0.031
20	15	10	1.00	1.95	0.042
20	15	5.2	0.72	2.07	0.062
20	15	99	2.00	1.79	0.097
20	15	50	1.70	1.90	0.092
20	15	21	1.32	2.06	0.091
20	15	10	1.00	2.20	0.098
30	25	5.2	0.72	2.34	0.111

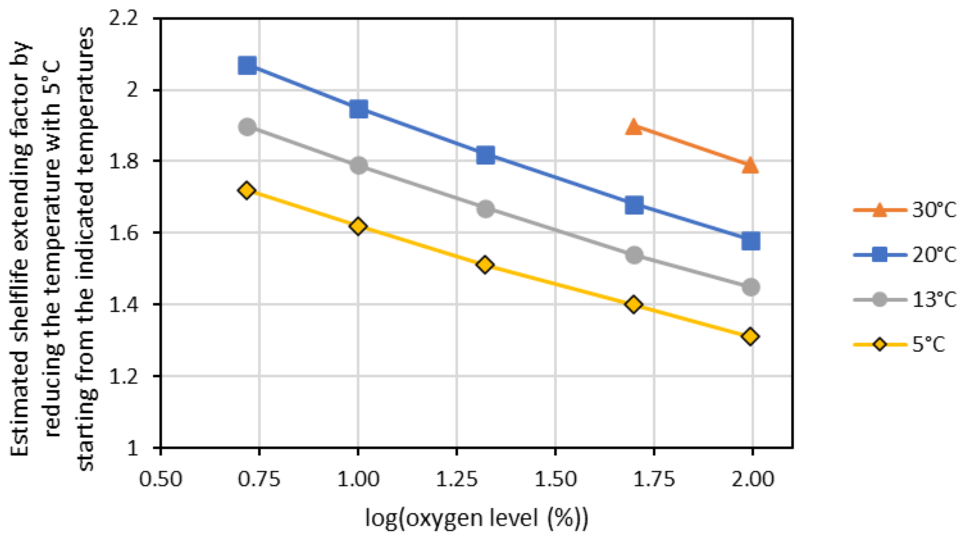

Table S5. Estimated p_{50} values in days for the different models for eRH^{20} 60%.
Values are according to a separate fitted curve for each storage combination, Model 0 for the E&R model with temperature *TEMP* and log*OXY* (= log(% oxygen)) only, and for Models 1, 2 and 3 the terms logOXY^2, *TEMP* x log*OXY*, $TEMP^2$ are added according to the forward selection. The RH in the storage jars was measured after equilibration at 20°C (eRH^{20}). The actual eRH during storage will have deviated slightly, see Table 1.

TEMP (°C)	*OXY* (%)	*Separate*	*Start* *Model 0*	+ logOXY^2 *Model 1*	+ *TEMP* x log*OXY* *Model 2*	+ $TEMP^2$ *Model 3*
5	5.2	652	570	477	565	614
5	10	533	407	431	470	510
5	21	347	277	327	325	350
5	50	148	177	189	169	180
5	99	94	125	104	86	90
13	5.2	141	199	166	168	155
13	10	143	142	150	150	140
13	21	117	97	114	113	105
13	50	67	62	66	65	60
13	99	32	44	36	36	33
20	5.2	50	79	66	58	53
20	10	46	57	60	55	51
20	21	38	39	45	45	42
20	50	26	25	26	28	26
20	99	17	17	14	17	15
30	5.2	16	21	18	13	14
30	10	15	15	16	13	15
30	21	15	10	12	12	14
30	50	8	7	7	9	10
30	99	6	5	4	6	7

Table S6. Estimated shelf life extension factors (ratio + standard errors) at different temperatures by halving the oxygen level for Model 3 at eRH^{20} level 60%. The RH in the storage jars was measured after equilibration at 20°C (eRH^{20}). The actual eRH during storage will have deviated slightly, see Table 1.

oxygen (%)	Halving the oxygen level	temperature (°C)	ratio	s.e. ratio
99	49.5	5	2.02	0.070
50	25.0	5	1.74	0.035
21	10.5	5	1.43	0.030
10	5.0	5	1.21	0.045
5.2	2.6	5	1.05	0.056
99	49.5	13	1.85	0.062
50	25.0	13	1.59	0.027
21	10.5	13	1.31	0.021
10	5.0	13	1.11	0.038
5.2	2.6	13	0.96	0.049
99	49.5	20	1.72	0.061
50	25.0	20	1.47	0.029
21	10.5	20	1.21	0.022
10	5.0	20	1.03	0.036
5.2	2.6	20	0.89	0.046
99	49.5	30	1.54	0.065
50	25.0	30	1.32	0.039
21	10.5	30	1.09	0.030
10	5.0	30	0.92	0.037
5.2	2.6	30	0.80	0.044

Table S7. Estimated shelf life extension factors (ratio + standard errors) at different oxygen levels by dropping the temperature by 5°C for Model 3 at eRH^{20} level 60%. The RH in the storage jars was measured after equilibration at 20°C (eRH^{20}). The actual eRH during storage will have deviated slightly, see Table 1.

Temperature (°C)	5 °C cooler	Oxygen (%)	ratio	s.e. ratio
5	0	99	2.03	0.081
5	0	50	2.14	0.081
5	0	21	2.30	0.086
5	0	10	2.43	0.094
5	0	5.2	2.56	0.105
13	8	99	1.84	0.044
13	8	50	1.94	0.040
13	8	21	2.08	0.039
13	8	10	2.20	0.045
13	8	5.2	2.32	0.056
20	15	99	1.69	0.034
20	15	50	1.78	0.026
20	15	21	1.91	0.021
20	15	10	2.02	0.026
20	15	5.2	2.13	0.037
30	25	99	1.49	0.052
30	25	50	1.58	0.050
30	25	21	1.69	0.050
30	25	10	1.79	0.054
30	25	5.2	1.88	0.060

Table S8. Analysis of deviance for the combined E&R model for eRH^{20} levels 16, 33 and 43%. Forward selection starts with the model with *RH*, *TEMP*, log*OXY*. The RH in the storage jars was measured after equilibration at 20°C (eRH^{20}). The actual eRH during storage will have deviated slightly, see Table 1.

Terms	Combined Model	Deviance	Mean Deviance	Deviance Difference	F-value	P-value
Start	C0	13877.93	26.38	-	-	-
+ RH^2	C1	12584.17	23.97	1293.77	53.97	< 0.001
+ *logOXY* x *RH*	C2	12286.37	23.45	297.80	12.70	< 0.001
+ *TEMP* x *logOXY*	C3	12257.78	23.44	28.59	1.22	0.270
+ $TEMP^2$	C4	12230.18	23.43	27.60	1.18	0.278
+ $logOXY^2$	C5	12225.81	23.47	4.36	0.19	0.667
+ *TEMP* x *RH*	C6	12225.81	23.51	0.00	0.00	0.999

Modelling the quantitative effect of oxygen on the ageing of primed celery seeds
Supporting Figures and Tables

Table S9. Estimated p_{50} values in days for the different models for the combined eRH^{20} levels 16, 33 and 43%. Values according to a separate fitted curve for each storage combination, Model C0 for the E&R model with temperature eRH^{20}, *TEMP* and log*OXY* (= log(% oxygen)) only, and for Models C1 and C2 the terms eRH^2 and log*OXY* x *RH* are added according to the forward selection. The RH in the storage jars was measured after equilibration at 20°C (eRH^{20}). The actual eRH during storage will have deviated slightly, see Table 1.

eRH^{20}	*TEMP*	*OXY*	*Separate*	*Start Model C0*	*+ RH^2 Model C1*	*+ logOXY x RH Model C2*
16	5	5.2	1697	3036	3181	2834
16	5	10	1880	1824	1903	1777
16	5	21	1297	1023	1063	1046
16	5	50	718	520	538	563
16	5	99	325	306	314	346
16	13	5.2	1165	1256	1332	1193
16	13	10	807	755	797	748
16	13	21	443	423	445	440
16	13	50	223	215	225	237
16	13	99	137	126	132	146
16	20	5.2	544	580	622	559
16	20	10	318	349	372	351
16	20	21	193	196	208	206
16	20	50	112	99	105	111
16	20	99	76	58	61	68
16	30	5.2	194	193	210	190
16	30	10	111	116	125	119
16	30	21	70	65	70	70
16	30	50	39	33	35	38
16	30	99	13	19	21	23
33	5	5.2	1782	2119	1781	1797
33	5	10	1177	1273	1066	1066
33	5	21	536	714	595	590
33	5	50	272	363	301	295
33	5	99	153	213	176	171
33	13	5.2	705	877	746	756
33	13	10	448	527	446	449
33	13	21	195	295	249	248
33	13	50	115	150	126	124
33	13	99	72	88	74	72
33	20	5.2	323	405	348	355
33	20	10	182	243	208	210
33	20	21	103	137	116	116
33	20	50	62	69	59	58
33	20	99	26	41	34	34
33	30	5.2	148	134	117	120
33	30	10	88	81	70	71
33	30	21	50	45	39	39
33	30	50	23	23	20	20

Table S9 continued. Estimated p_{50} values in days for the different models for the combined eRH[20] levels 16, 33 and 43%. Values according to a separate fitted curve for each storage combination, Model C0 for the E&R model with temperature *eRH[20]*, *TEMP* and log*OXY* (= log(% oxygen)) only, and for Models C1 and C2 the terms *eRH²* and log*OXY* x *RH* are added according to the forward selection. The RH in the storage jars was measured after equilibration at 20°C (eRH[20]). The actual eRH during storage will have deviated slightly, see Table 1.

eRH[20]	*TEMP*	*logOXY*	*Separate*	*Start Model C0*	*+ RH² Model C1*	*+ logOXY x RH Model C2*
33	30	99	14	14	12	11
43	5	5.2	2836	1689	1854	2038
43	5	10	1458	1015	1110	1168
43	5	21	671	569	620	622
43	5	50	243	290	313	297
43	5	99	132	170	183	166
43	13	5.2	989	699	777	858
43	13	10	582	420	465	492
43	13	21	249	236	259	262
43	13	50	102	120	131	125
43	13	99	88	70	77	70
43	20	5.2	364	323	363	402
43	20	10	229	194	217	231
43	20	21	134	109	121	123
43	20	50	69	55	61	59
43	20	99	49	33	36	33
43	30	5.2	64	107	122	136
43	30	10	55	64	73	78
43	30	21	33	36	41	42
43	30	50	19	18	21	20
43	30	99	12	11	12	11

Table S10. Analysis of deviance for the combined E&R model for eRH^{20} levels 16, 33 and 43% employing RH according to the Cromarty equation (*CRH*). Forward selection starts with the model with *CRH*, *TEMP*, log*OXY*.

Terms	Combined Model	Deviance	Mean Deviance	Deviance Difference	F-value	P-value
Start	C0	13944.76	26.51	-	-	-
+ CRH^2	C1	13210.11	25.16	734.65	29.20	< 0.001
+ *TEMP* x *CRH*	C2	13010.72	24.83	199.39	8.03	0.005
+ *logOXY* x *CRH*	C3	12837.24	24.55	173.49	7.07	0.008
+ *TEMP* x *logOXY*	C4	12743.11	24.41	94.13	3.86	0.050
+ $TEMP^2$	C5	12691.17	24.36	51.94	2.13	0.145
+ $logOXY^2$	C6	12685.31	24.39	5.86	0.24	0.624
