# Supplementary material for: Modelling the quantitative effect of oxygen on the ageing of primed celery seeds
Source: Plant J. 2025 Apr 17;122(1):e70066. doi: 10.1111/tpj.70066 (PMC12005833; doi:10.1111/tpj.70066)
Supplement: Supplementary file 4 — Appendix S4. Formula for the shelf life extension factor for the E&R model with several covariates. [file TPJ-122-0-s003.pdf]

Modelling the quantitative effect of oxygen on the ageing of primed celery seeds

Supporting Appendix S2. Formula for the shelf life extension factor for the E&R model with several covariates

The E&R viability model (Ellis and Roberts, 1980) is given by

$$\nu = K_i - p/\sigma_S \tag{1}$$

in which ν denotes the probit of the viability, K_i is the probit of the initial viability at zero storage days, p is the storage time in days, and σ_S is the time for viability to fall by one probit, which depends on the storage conditions S. The time at which a percentage Q of the seeds is still alive for storage conditions S, denoted by $p_Q(S)$, is then given by

$$p_Q(S) = \sigma_S\ [K_i - probit(Q)] \tag{2}$$

The so-called shelf life extension factor E_{12} for replacing storage condition S_1 by condition S_2 is defined by the ratio of their p_Q values, i.e.

$$E_{12} = p_Q(S_2)/p_Q(S_1) = \sigma_{S_2}/\sigma_{S_1} \tag{3}$$

This reveals that the extension factor does not depend on the initial viability K_i, nor on the percentage Q.

In our experiment σ_S may depend on temperature (T), oxygen (O) on a logarithmic scale and relative humidity (H). A general E&R model with these three terms, their quadratic effects, interactions and parameters C_0 … C_9 is given by

$$\sigma_S = 10^{\,C_0 - C_1T - C_2\,log(O) - C_3H - C_4T^2 - C_5\,log^2(O) - C_6H^2 - C_7T\ log(O)\ - C_8TH - C_9H\,log(O)} \tag{4}$$

Note that for a model with a fixed level of the relative humidity $C_3 = C_6 = C_8 = C_9 = 0$. Shelf life extension factors can be calculated by combining eq. (3) and (4).

The extension factor $E_{T_{12}}$ for **replacing temperature T_1 by T_2** for fixed values of O and H is given by

$$E_{T_{12}} = 10^{-[C_1 + C_7\,log(O) + C_8H]\,(T_2 - T_1)\, -\, C_4\,(T_2^2 - T_1^2)} \tag{5}$$

The extension factor $E_{O_{12}}$ for **replacing oxygen level O_1 by O_2** for fixed values of T and H is given by

$$E_{O_{12}} = 10^{\,-[C_2 + C_7T + C_9H]\,(log(O_2) - log(O_1)) - \,C_5\,(log^2(O_2)\, -\, log^2(O_1))} \tag{6}$$

For the special case $C_5 = C_7 = C_9 = 0$, the extension factor $E_{O_{12}} = 10^{-C_2\,(log(O_2) - log(O_1))}$. This implies that halving the oxygen level gives an extension factor of 0.5^{-C_2}. Likewise, for the special case $C_5 = 0$, halving the oxygen level gives $E_{O_{12}} = 0.5^{-[C_2 + C_7T + C_9H]}$.

The extension factor $E_{H_{12}}$ for **replacing humidity H_1 by H_2** for fixed values of T and O is given by

$$E_{H_{12}} = 10^{-[C_3 + C_8T + C_9\,log(O)]\,(H_2 - H_1) - \,C_6\,(H_2^2 - H_1^2)} \tag{7}$$

Given estimates of the C parameters and the associated variance-covariance matrix, these equations provide estimates of the ratios. Standard errors of the ratios can be calculated by the delta method (Oehlert, 1992).

Modelling the quantitative effect of oxygen on the ageing of primed celery seeds

References

Ellis, R. H., & Roberts, E. H. (1980). Improved equations for the prediction of seed longevity. *Annals of Botany, 45*, 13-30. https://doi.org/10.1093/oxfordjournals.aob.a085798.

Oehlert, G. W. (1992). A Note on the Delta Method. *The American Statistician, 46 (1)*, 27-29. https://www.jstor.org/stable/2684406.
